# A Viral Genome Landscape of RNA Polyadenylation from KSHV Latent to Lytic Infection

**DOI:** 10.1371/journal.ppat.1003749

**Published:** 2013-11-14

**Authors:** Vladimir Majerciak, Ting Ni, Wenjing Yang, Bowen Meng, Jun Zhu, Zhi-Ming Zheng

**Affiliations:** 1 Tumor Virus RNA Biology Section, Gene Regulation and Chromosome Biology Laboratory, Center for Cancer Research, National Cancer Institute, National Institutes of Health, Bethesda, Maryland, United States of America; 2 DNA Sequencing and Genomics Core, National Heart, Lung, and Blood Institute, National Institutes of Health, Bethesda, Maryland, United States of America; University of California Berkeley, United States of America

## Abstract

RNA polyadenylation (pA) is one of the major steps in regulation of gene expression at the posttranscriptional level. In this report, a genome landscape of pA sites of viral transcripts in B lymphocytes with Kaposi sarcoma-associated herpesvirus (KSHV) infection was constructed using a modified PA-seq strategy. We identified 67 unique pA sites, of which 55 could be assigned for expression of annotated ∼90 KSHV genes. Among the assigned pA sites, twenty are for expression of individual single genes and the rest for multiple genes (average 2.7 genes per pA site) in cluster-gene loci of the genome. A few novel viral pA sites that could not be assigned to any known KSHV genes are often positioned in the antisense strand to ORF8, ORF21, ORF34, K8 and ORF50, and their associated antisense mRNAs to ORF21, ORF34 and K8 could be verified by 3′RACE. The usage of each mapped pA site correlates to its peak size, the larger (broad and wide) peak size, the more usage and thus, the higher expression of the pA site-associated gene(s). Similar to mammalian transcripts, KSHV RNA polyadenylation employs two major poly(A) signals, AAUAAA and AUUAAA, and is regulated by conservation of *cis*-elements flanking the mapped pA sites. Moreover, we found two or more alternative pA sites downstream of ORF54, K2 (vIL6), K9 (vIRF1), K10.5 (vIRF3), K11 (vIRF2), K12 (Kaposin A), T1.5, and PAN genes and experimentally validated the alternative polyadenylation for the expression of KSHV ORF54, K11, and T1.5 transcripts. Together, our data provide not only a comprehensive pA site landscape for understanding KSHV genome structure and gene expression, but also the first evidence of alternative polyadenylation as another layer of posttranscriptional regulation in viral gene expression.

## Author Summary

A genome-wide polyadenylation landscape in the expression of human herpesviruses has not been reported. In this study, we provide the first genome landscape of viral RNA polyadenylation sites in B cells from KSHV latent to lytic infection by using a modified PA-seq protocol and selectively validated by 3′ RACE. We found that KSHV genome contains 67 active pA sites for the expression of its ∼90 genes and a few antisense transcripts. Among the mapped pA sites, a large fraction of them are for the expression of cluster genes and the production of bicistronic or polycistronic transcripts from KSHV genome and only one-third are used for the expression of single genes. We found that the size of individual PA peaks is positively correlated with the usage of corresponding pA site, which is determined by the number of reads within the PA peak from latent to lytic KSHV infection, and the strength of *cis*-elements surrounding KSHV pA site determines the expression level of viral genes. Lastly, we identified and experimentally validated alternative polyadenylation of KSHV ORF54, T1.5, and K11 during viral lytic infection. To our knowledge, this is the first report on alternative polyadenylation events in KSHV infection.

## Introduction

Kaposi sarcoma-associated herpesvirus (KSHV), also referred as human herpesvirus 8 (HHV-8), is a member of gammaherpervirus subfamily [1]. KSHV infection in healthy individuals is well-controlled by host immune system and other host factors, and hence, it is usually asymptomatic. However prolonged immunosuppression may lead to occurrence of KSHV-induced malignancies. KSHV has been linked to three malignancies including all forms of Kaposi sarcoma, a complex solid tumor of endothelial origin, and two rare B-cell lymphomas, primary effusion lymphoma (PEL or body cavity-based large cell lymphoma) and multicentric Castleman disease [2]–[4]. KSHV, like other herpesviruses, exhibits two distinguishable states of infection, latent and lytic infection. During latency only a small fraction of viral genes are expressed to facilitate the maintenance of viral genome, drive cell proliferation, and mediate immune invasion. Various external and internal stimuli cause a disruption of KSHV latency and induction of KSHV lytic infection with the expression of all viral lytic genes and replication of viral progeny [5]–[7]. KSHV has a large DNA genome (∼168 kb) encoding more than 90 genes for production of viral structural and non-structural proteins, small peptides, long non-coding RNAs (lncRNAs) and small regulatory miRNAs [1], [8]–[10]. Like many DNA viruses, KSHV has a complex gene organization and depends on host cell machinery for its gene expression. However, a full compendium of viral genome annotation is still unknown and the true nature of viral gene expression and its regulation remains to be fully understood.

RNA polyadenylation (pA) of nascent transcripts is a critical posttranscriptional step in maturation of eukaryotic transcripts [11]. A primary role of RNA polyadenylation is to release newly synthesized RNA from DNA template through endonuclease cleavage and protect it from degradation by addition of a poly(A) tail to the RNA 3′ end. The presence of a poly(A) tail also promotes nucleocytoplasmic export and efficient protein translation of mRNAs [12], [13]. RNA polyadenylation is carried out by a large protein complex composed of at least 85 protein factors and binds to specific sequences within nascent transcripts surrounding the cleavage site [14]. An A/U-rich element upstream, recognized by cleavage and polyadenylation specificity factor (CPSF), and an U/GU-rich element downstream, recognized by cleavage stimulatory factor (CstF) [15], [16], of the cleavage site are two major determinants of RNA polyadenylation, although other auxiliary *cis*-elements may be also involved in pA site definition [17], [18]. After assembly of polyadenylation complex the pre-mRNA is generally cleaved at “CA” dinucleotide followed by addition of a poly(A) tail [19]. While the process of polyadenylation itself is well characterized, the selection of pA site remains a puzzle. Recent genome-wide studies on polyadenylation of host transcripts in various organisms revealed highly promiscuous polyadenylation of large population of RNAs from multiple pA sites [20]–[22]. As a result, genes affected by alternative polyadenylation produce a subset of transcripts with different coding potentials or 3′ untranslated regions (3′ UTRs) [23].

A polyadenylation landscape of human herpesviruses has not been reported at the genome-wide level. In this study, we performed a genome-wide analysis on RNA polyadenylation of KSHV transcripts from B cells with latent or lytic viral infection by using a modified polyadenylation- sequencing (PA-seq) technology [24]. We identified that KSHV utilizes 67 active pA sites for the expression of its latent and lytic genes and a few novel or unannotated genes. We also found alternative RNA polyadenylation of several known KSHV genes and revealed pA site *cis*-elements in regulation of KSHV gene expression.

## Results

### Identification of KSHV pA sites by PA-seq

To elucidate a role of RNA polyadenylation in KHSV gene regulation, we performed a genome-wide analysis of viral pA sites to monitor their usage during KSHV infection. Three KSHV-positive B-cell lines (JSC-1, BCBL-1 and TREx BCBL-1), which support both latent and lytic virus infection, were chosen in this study. For each cell line, polyadenylation events were compared between latent and lytic infection (Figure S1A). The cells with lytic infection were harvested at 48 h after virus reactivation by chemical induction to allow full viral replication cycle and sufficient expression of viral late transcripts. We observed dramatic reduction of cell viability associated with virus reactivation (31% vs 88% for JSC-1, 50% vs 89% for BCBL-1 and 20% vs 82% for TREx BCBL-1 cells [further referred as TREx]) by trypan blue exclusion analysis. Poly (A)^+^ RNA fraction from each sample was used for preparing 3′-end cDNA libraries with a modified PA-seq method followed by *Illumina* paired-end sequencing [24], [25]. In total, we obtained more than 119 million of paired reads from all samples (Figure S1B). KSHV- and human-specific reads were extracted by alignment of obtained sequence reads to the reference KSHV (GenBank acc no U75698.1) and human (UCSC version hg19) genomes. More than 100 million (∼84%) of all reads were uniquely mapped, with about 35 million (∼29%) to KSHV and approximately 65 million (∼55%) to human genome. The remaining 19 million (16%) are unmapped reads. As expected, a remarkable correlation was noticed between KSHV-specific reads and the stat of KSHV infection in all three cell lines, with less KSHV reads (0.10–0.47%) in the cells with viral latent infection and much more KSHV reads (20–77%) in the cells with KSHV lytic infection (Figure S1B and S1C).

For KSHV pA site analysis we focused only on the sequence reads uniquely mapped to KSHV genome and further clustered to identify PA-peaks. We pooled KSHV sequence reads from all samples and performed a peak calling analysis using F-seq algorithm with significant enrichment over a background model [26] and a threshold of >50 read counts per peak (Figure S2). As expected, only a handful of PA-peaks were found in the cells with latent KSHV infection, but significantly more peaks were detected in the cells with lytic KSHV infection (Figure S3A). To further analyze the PA-peak distributions in the context of selected viral genes and to ensure the PA-peaks obtained from our PA-seq representative of authentic pA site regions, we looked into a PA-peak distributed in a well-characterized ORF50 (RTA)/K8/K8.1 locus which encodes three collinear KSHV genes. Although each gene in the locus has its own promoter, their transcripts are all polyadenylated at a single pA site downstream of K8.1 (see diagram in Figure S3B). We found a prominent PA-peak in all lytic samples, but less so in the latent samples, overlapping with the mapped pA site (Figure S3B) reported in previous studies [27], [28]. No other PA-peaks were seen either upstream or downstream of this pA site. These data indicate that PA-seq libraries were in high quality and suitable for comprehensive analysis of viral pA sites.

**Figure 1:**
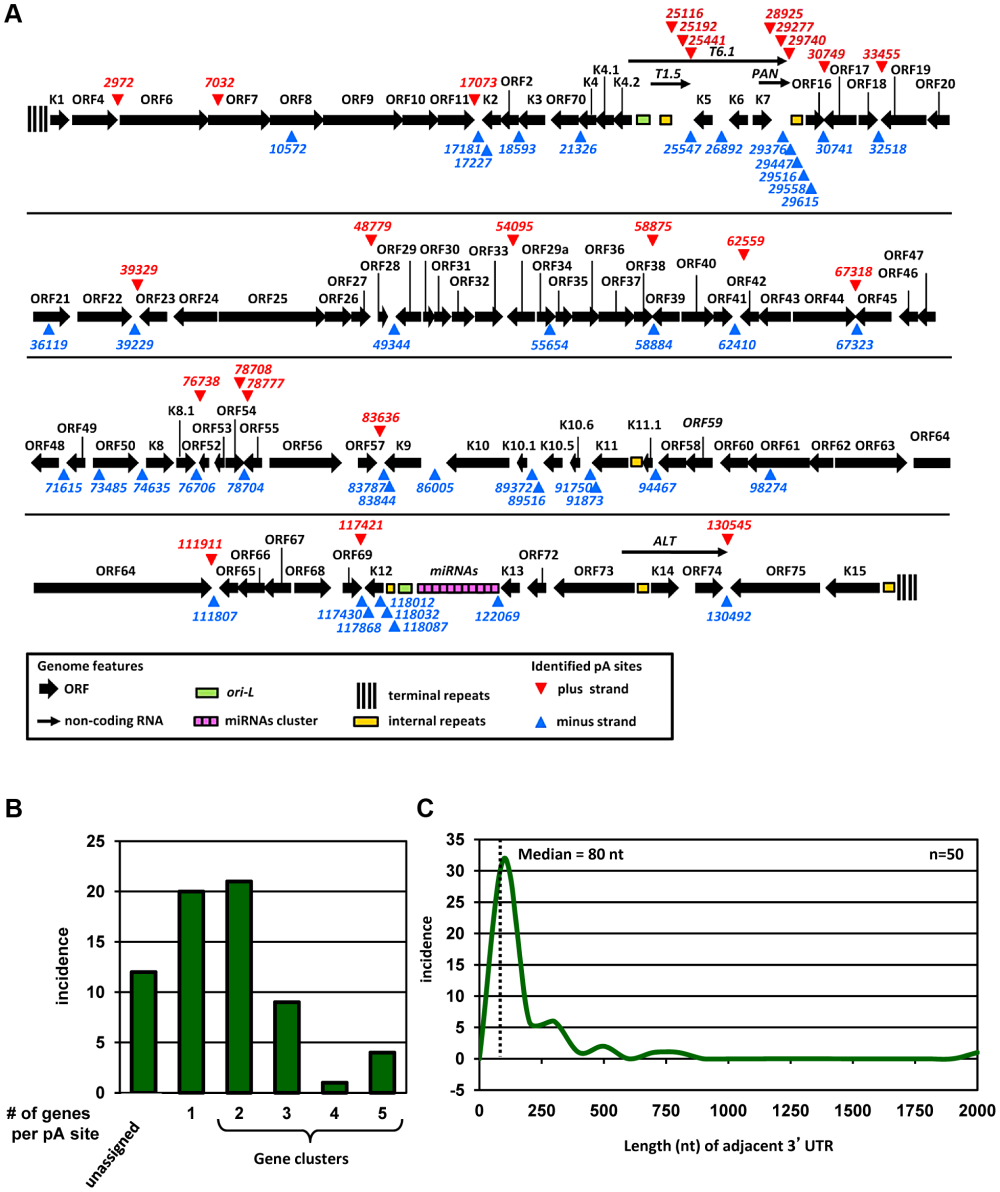
Genome-wide landscape of KSHV pA sites. (A) A diagram of KSHV genome with mapped viral pA sites (red triangles for plus strand and blue triangles for minus strand). Each numbers represents the nucleotide position of an identified pA site. (B) Incidence of pA sites mapped to single viral genes or in gene clusters (two or more genes per pA site). (C) Scatter plot depicting size distribution of viral 3′UTR length from the termination codon of a gene adjacent to the mapped pA site immediately downstream. Median 3′UTR length was calculated from 50 pA sites immediately downstream of protein coding ORFs.

Subsequently, we determined the nucleotide (nt) position with the highest number of reads within individual peaks in the peak calling analysis as a PA mode (Figure S2) and designated such a PA mode as an unique pA site (Table S1). We also determined a PA peak from the peak start to the peak end, and the total number of the reads within a PA peak was used to approximate the usage of a pA site (Figure S2). With this approach we identified 67 pA sites on both viral DNA strands of the KSHV genome (Figure 1A). The pA sites mapped by PA-seq in this study were remarkably close to several known pA sites previously mapped by traditional methods both in terms of mapped nucleotide position and strand specificity (Table S2).

A higher prevalence of pA sites shows strand bias, with 43 pA sites in the minus strand and 24 in the plus strand of the KSHV genome. The majority of the mapped pA sites are positioned in the intergenic regions of KSHV genome, outside of annotated ORFs, with exception of the pA sites in the coding regions of ORF7 at nt 7032 and ORF61 at nt 98274 and of K12 at nt 118012, 118032 and 118087.

### Assignment of mapped pA sites to KSHV genes

Our genome-wide pA site analysis allowed us to correlate each mapped pA site to annotated KSHV genes and to identify novel KSHV gene(s). We assigned each pA site to a known viral gene or gene cluster region based on the following criteria: (1) both of the gene(s) and the corresponding pA site must be on the same strand of viral genome, (2) the pA site must be positioned outside of the coding region of the viral gene(s), and (3) the gene(s) assigned to a mapped pA site must be positioned upstream of the pA site. These criteria assume that viral transcripts originated from a promoter(s) upstream of the gene will be polyadenylated from the first available pA site downstream. Accordingly, we assigned 55 pA sites to all known KSHV genes (Figure 1B, Table S3). The remaining 12 pA sites unable to assign would indicate the presence of transcripts from unknown KSHV genes for further validation. Interestingly, the majority of unassigned pA sites are positioned antisense to known KSHV genes, suggesting the existence of putative antisense transcripts to these viral genes [29]. Among 55 pA sites assigned to known KSHV gene transcripts, 20 are positioned immediately downstream of a single KSHV gene for polyadenylation of a monocistronic mRNA, and the remaining 35 have multiple upstream KSHV genes ranging from 2 to 5 for polyadenylation of bicistronic or polycistronic transcripts (Figure 1B). Interestingly, we found two or more pA sites mapped to a region downstream of the same gene. These include two alternative pA sites downstream of ORF54, K2 (vIL6), K9 (vIRF1), K10.5 (vIRF3), K11 (vIRF2), and K12 (Kaposin A), three downstream of T1.5 RNA and PAN (nut-1) RNA or in an internal K12 region, and five downstream of *vnct* internal repeats (Figure 1A). From protein-coding genes, ORF54 showed the highest usage of alternative pA sites (∼24%) followed by K10.5 (∼17%) and K11 (∼11%), but K2, K9, and K12 did so much less frequently (Table S4). Thus, our analyses provide not only the first comprehensive landscape of functional pA sites in the context of KSHV genome, but for the first time the alternative polyadenylation of KSHV transcripts during virus infection.

### The 3′UTR length of KSHV transcripts

We next aimed to determine the length and composition of 3′ UTR for each KSHV protein-coding gene. The unassigned pA sites and the pA site for viral non-coding RNA genes were excluded from this analysis. A total of 50 pA sites were used to calculate the 3′UTR length from a pA site to the adjacent termination codon of the closest upstream ORF. We found that the calculated 3′ UTR length of KSHV genes varies greatly in size from 2 nts (ORF38) to 1925 nts (ORF62) (Table S3). The distribution of KSHV 3′UTR is shown in Figure 1C, with a median size of the 3′UTR in ∼80 nts which is significantly shorter than human 3′ UTR with a median size of ∼300 nts [20].

### Usage of KSHV pA sites during KSHV life cycle

**Figure 2:**
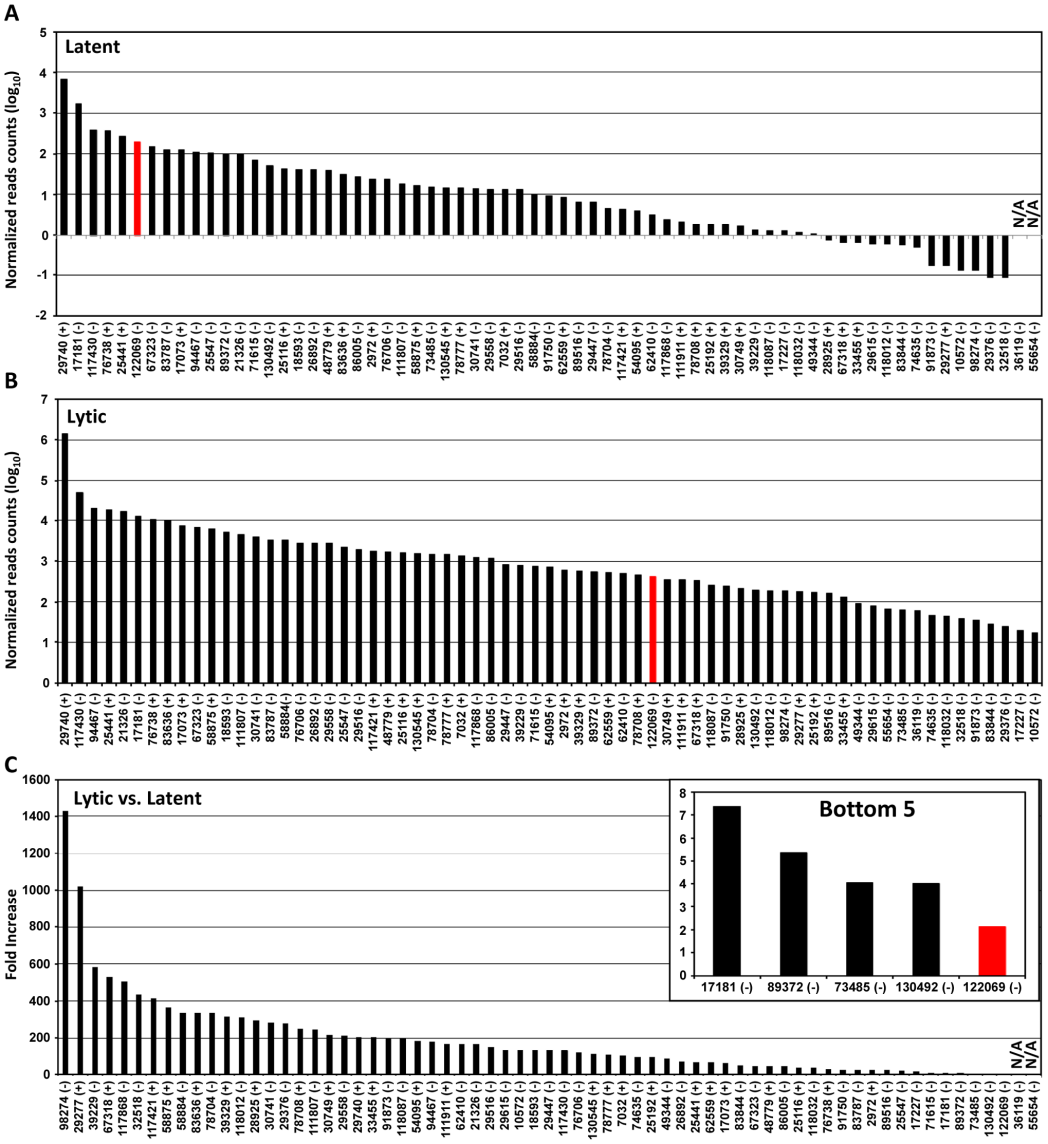
Usage of identified KSHV pA sites from latent to lytic infection. (A and B) Bar graphs representing frequency of each identified pA site usage from all 3 samples with latent (A) or lytic (B) infection after normalization to per million of all mapped reads. (C) The bar graph showing a fold change in each pA site usage from lytic (A) to latent (B) infection. The inset shows bottom five pA sites with the lowest change during lytic infection. The red bars in (A to C) represent a previously reported pA site of a KSHV latent transcript, ORF73/ORF72/K13. N/A, not applicable.

**Figure 3:**
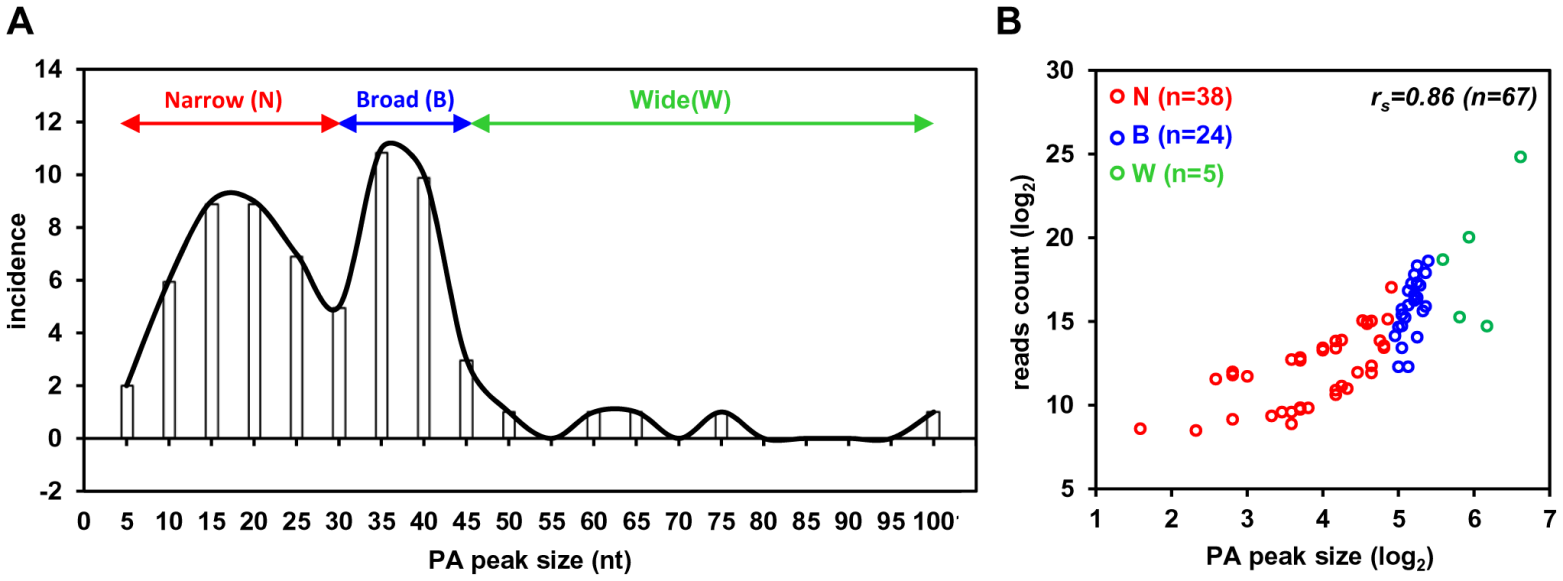
Peak size and usage of KSHV pA sites. (A) A plot showing a distribution of identified viral pA sites based on the PA peak size determined by F-seq analysis. All pA sites are divided into three categories based on their peak size: narrow (≤30 nts), broad (>30, ≤45 nts) and wide (>45 nts). (B) Scatter plot depicting correlation between PA peak sizes (x-axis) and their usage (y-axis). Each color circle represents a mapped pA site. The Spearman correlation coefficient (*r_s_*) was calculated from all viral pA sites.

Based on the number of sequence reads obtained at each pA site, one can infer the relative steady-state level (pA site usage) of the pA site-associated transcripts. The limitation of this approach is cluster genes utilizing a single pA site, in which the number of sequence reads reflects a combined level of all gene transcripts. The pA site usage was compared from latent to lytic infection. First, we determined each pA site usage in individual samples to obtain a sample-specific pA site usage and then normalized the number of sequence reads within each viral pA peak to the total sequence reads mapped to KSHV and host genome in each sample (Figure S4, Table S5). Combination of the normalized sequence reads from all latent samples was compared with that from all lytic samples (Figure 2A, 2B, Table S6). The pA site 122069 (+) of latent polycistronic RNA ORF73 (LANA), ORF72 (vCyclin), and K13 (vFLICE) was served as a reference (red bar in Figure 2A–C). Surprisingly in the samples with latent infection, the top 5 sites based on the pA site sequence counts were PAN (nut-1), ORF2/K2 cluster, K12, ORF50/K8/K8.1 cluster, and T1.5 (Figure 2A) which supposed to be KSHV lytic genes, but spontaneously reactivated in a small fraction of cells with latent infection (Figure S3, Table S6). The usage of pA site for the expression of ORF73/ORF72/K13 ranked the 6th during the latency. In the samples with lytic infection, the top 5 pA site usage was the pA sites for abundant expression of PAN, K12, ORF62-58 cluster, T1.5, and K4.2/4.1/4 cluster and usage of the reference latent pA site for expression of ORF73/ORF72/K13 dropped to the 41st. When the changes in utilization of each identified pA site from lytic to latent infection were calculated, however, the usage of mapped pA sites for virus lytic gene expression became remarkable, with more than 500-fold increase from latent to lytic infection for ORF62, ORF24/23, ORF44, PAN, and K12 (Figure 2C, Table S6). The smallest usage change (<10 fold) during virus lytic infection was the pA sites for the expression of ORF2/K2, K10.6/10.5, and ORF73/ORF72/K13, and the transcripts antisense to ORF50 (RTA) and K15/ORF75 (Figure 2C, inset). The smallest change in the pA site usage was the reference pA site of ORF73/ORF72/K13, with only 2.1-fold increase. Thus, these pA sites are truly used to express viral latent genes. Notably, the peak sizes of pA sites vary considerably ranging from 3 (pA site at nt 17227) to 98 nts (pA site at 29740) (Table S7), and represents heterogeneity of the cleavage sites within each mapped pA site [30]. This heterogeneity of a given pA site, such as the pA site at nt 29740, nt 76738 or nt 122069, remained invariable either from JSC-1 to BCBL-1 cells or from latent to lytic infection (data not shown). Based on their bimodal distribution (Figure 3A), we grouped 38 pA sites with a narrow peak (≤30 nts, with a median size of 17 nts), 24 pA sites with a broad peak (>30, ≤45 nts, with a median size of 36.5 nts), and 5 pA sites with a wide peak (>45 nts, with a median size 61 nts) (Figure 3A). Interestingly, we found a strong positive correlation of pA site usage by sequence reads in the order from narrow (4,013 reads), broad (62,764 reads), to wide (425,475 reads) peaks with Spearman correlation coefficient *r_s_* = 0.86 (Figure 3B, Table S8). Because each transcript could produce only one read in PA-seq, whereas RNA-seq relies on the read coverage on the entire region of a transcript, the read count in a given PA peak of a pA site simply reflects the abundance of the corresponding transcript.

### RNA *cis*-elements in regulation of viral polyadenylation

**Figure 4:**
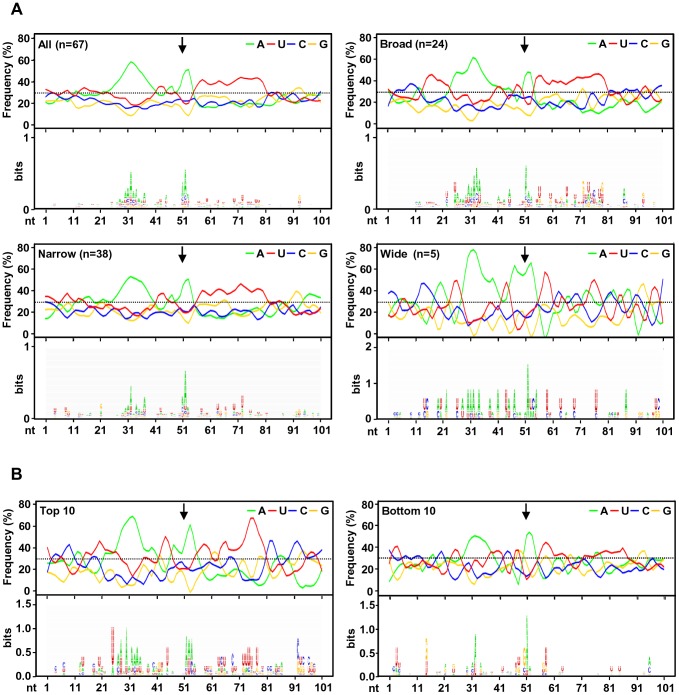
A sequence landscape surrounding KSHV pA sites. (A) Frequency (%) of each A, U, C, G (upper part of each panel) in the region ±50 nts of the mapped pA sites (arrows) was calculated either from all mapped pA sites or a subgroup of mapped narrow, broad or wide pA sites. The lower part of each panel represents motifs identified by Weblogo. (B) Nucleotide conservation in the same region of highly used top 10 and less used bottom 10 pA sites.

To investigate the regulatory elements responsible for polyadenylation of KSHV viral transcripts, we analyzed flanking sequences (±50 nts) of all 67 pA sites identified. Prevalence of each nucleotide at individual position was calculated and followed by motif analysis using WebLogo software (Figure 4A). A high prevalence of “A” residues between 10 to 30 nts upstream of the cleavage site was identified, representing the upstream A/U-rich polyadenylation signal. The cleavage site itself was also enriched in A residues, followed by a ∼30 nt long, mostly U-rich element. This distribution of RNA *cis*-elements around viral pA sites is in agreement with what has been found in human transcripts [16]. To better understand the role of *cis*-elements in regulation of KSHV polyadenylation, we performed similar analyses separately for three groups of pA sites with a narrow, broad, or wide peak (Figure 4A). The profiles of pA sites with a narrow and broad peak showed the highest similarity to the canonical pA site, with a defined upstream A-rich and a downstream U-rich element. The pA sites with a broad peak also exhibit a U-rich region further upstream. However, there is no significant U-rich element downstream of the pA site with a wide peak, nor other sequence motifs could be seen. These differences in sequence context surrounding the pA sites with different peaks could devote to their notable abundance of the associated transcripts, and was further reiterated by analysis of top 10 pA sites with the highest numbers of sequence reads and bottom 10 pA sites with the lowest number of sequence reads among all 67 pA sites. As shown in Figure 4B, the top 10 pA sites show highly conserved polyadenylation signals upstream and an U-rich region downstream. In contrast, the bottom 10 pA sites only exhibit less conserved polyadenylation signals and lack an U-rich region downstream.

**Figure 5:**
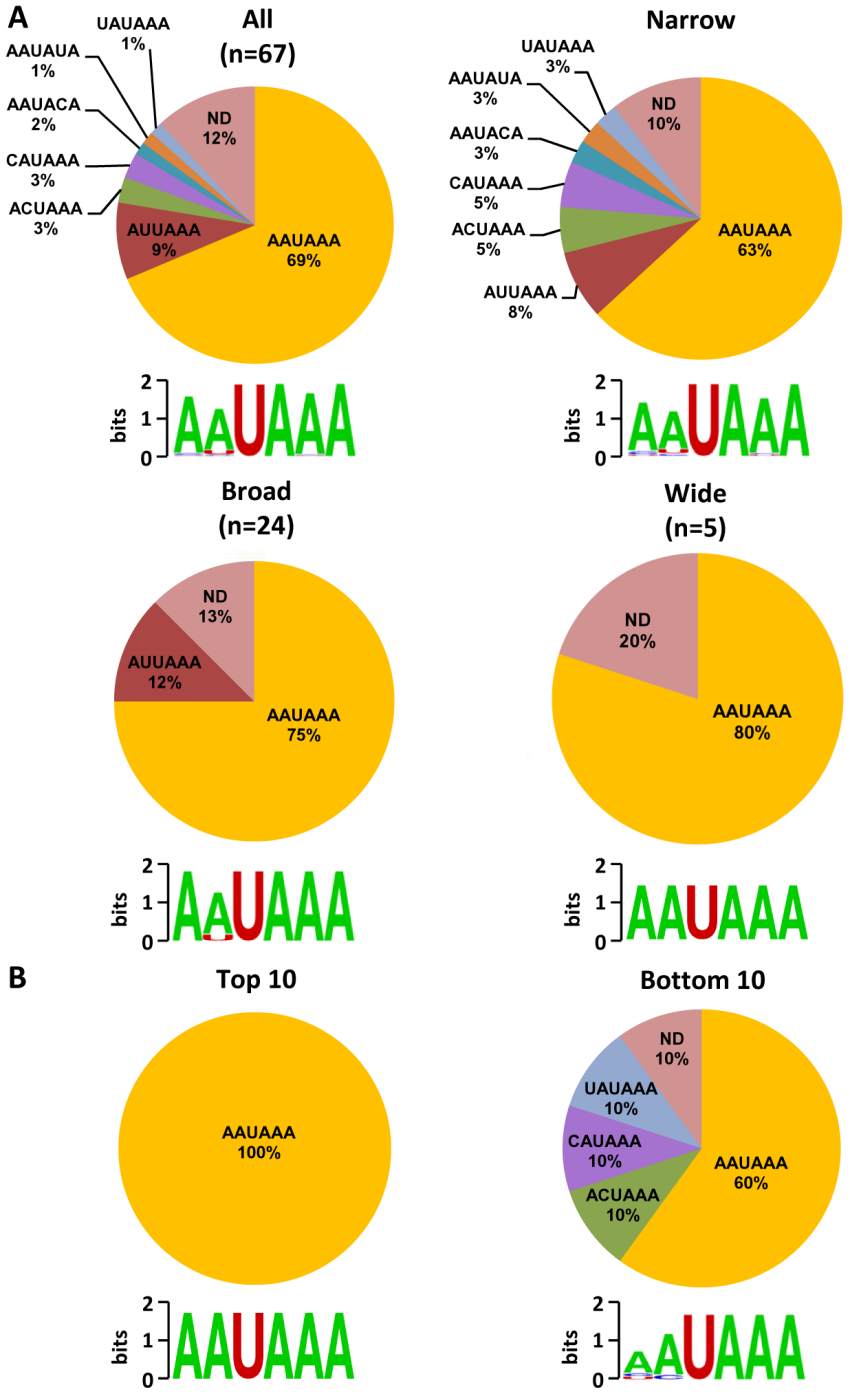
Poly (A) signal (PAS) and viral RNA polyadenylation. A region 50(AAUAAA) or non-canonical PAS. Pie charts showing percentage of each PAS identified in all mapped pA sites or in a subgrouped pA sites (narrow, broad or wide) (A) and in top 10 highly used and bottom 10 less used pA sites (B). ND, non-detectable. Diagrams below represent nucleotides conservation in identified PAS generated by Weblogo.

Analysis of upstream poly(A) signal (PAS) strength of KSHV pA sites further reaffirmed this conclusion. The canonical (AAUAAA) and non-canonical (NNAUNA) PAS were identified within 50 nts upstream of the mapped 59 pA sites (Figure 5A, Table S9). Two most common PAS in KSHV as seen in human polyadenylation are canonical AAUAAA (69%) followed by AUUAAA (9%). The usage of other non-canonical PAS for viral RNA polyadenylation ranges from 1% to 3% (Figure 5A, Table S10). Similar to human transcripts [20], about 12% of pA sites mapped in this study have no PAS. Surprisingly, we found that most of the non-canonical PAS were associated with a narrow peak and low level of expression. In contrast, the broad and wide peaks use predominantly canonical AAUAAA and AUUAAA PAS. This became even more obvious with PAS in the top and bottom used 10 pA sites. We found that all top 10 pA sites, but only 60% of the bottom 10 pA sites, contain the canonical AAUAAA (Figure 5B, Table S10).

### Experimental validation of selected KSHV pA sites

**Figure 6:**
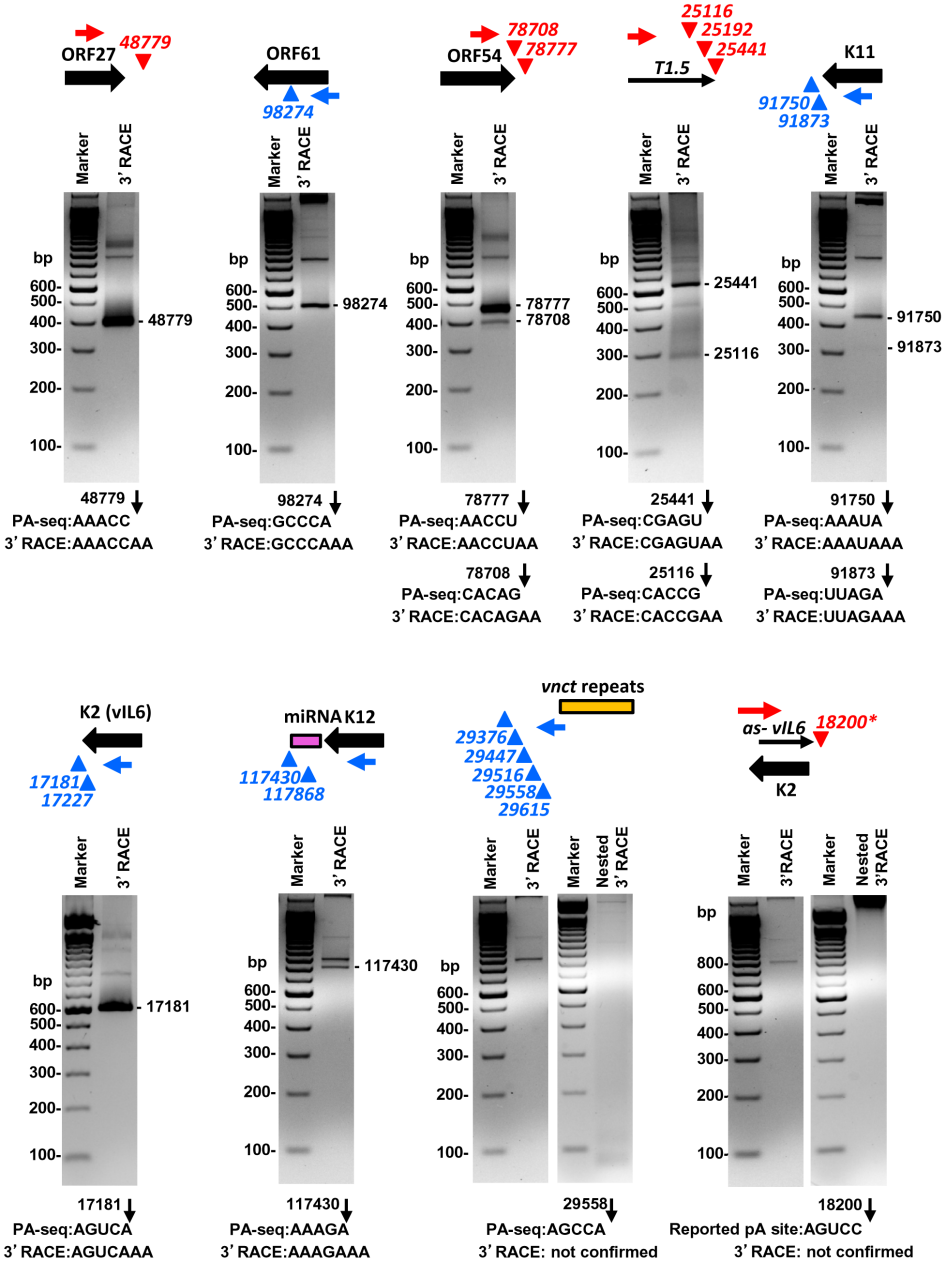
Validation of selected viral pA sites by 3′ RACE. Diagrams above each gel display transcription direction of a gene with the mapped pA site(s) in plus (red) or minus (blue) strand. Below each diagram are 3′RACE products from amplification by each gene-specific oligo (Supplemental Table S11) of total RNA extracted from TREx-RTA cells induced with doxycyline for 48 h. The sequence comparison of the mapped pA site(s) determined by PA-seq and 3′RACE are shown below each agarose gel, with numbers indicating the nucleotide positions of the mapped pA sites (black arrows) in the KSHV genome.

Given that the pA sites obtained by PA-seq, in general, showed a high correlation with previously mapped KSHV pA sites, we carried out a series of experiments to reconfirm several novel pA sites discovered in this study by 3′ RACE. These include the pA site downstream of ORF27, the pA site mapped within the coding region of ORF61, the alternative pA sites downstream of ORF54 and T1.5, and a cluster of 5 unassigned pA sites downstream of the *vnct* internal repeats, in addition to the known pA sites and unknown alternative pA sites of K11, K2/vIL6, and K12. Most of the selected pA sites determined by PA-seq were verified by sequencing the expected 3′ RACE products in the predicted size(s) (Figure 6, Table S11). The alternative pA site at nt 25192 within T1.5 lncRNA was not experimentally confirmed because of its <1% usage among T1.5 transcripts and lack of a searchable PAS upstream, nor the alternative pA sites at nt 17227 for K2/vIL6 and at nt 117868 for K12 because of their lower level usage. We were also unable to detect any 3′RACE product in the predicted sizes from five pA sites downstream of *vnct* internal repeats, despite their moderate usage based on the number of associated read counts. These pA sites identified by PA-seq are in proximity to the short internal 13-bp repeats region “*vnct*” between nt 29775 and nt 29942 of the KSHV genome. It is worth noting that four of the five pA sites have no detectable PAS upstream and a pA site at the nt 29615 has a non-canonical AAUAUA PAS. A reported pA site at nt 18200 for an RNA antisense to K2 (vIL6) [29], [31] which was not revealed by PA-seq was also not detectable by 3′ RACE in this study (Figure 6).

**Figure 7:**
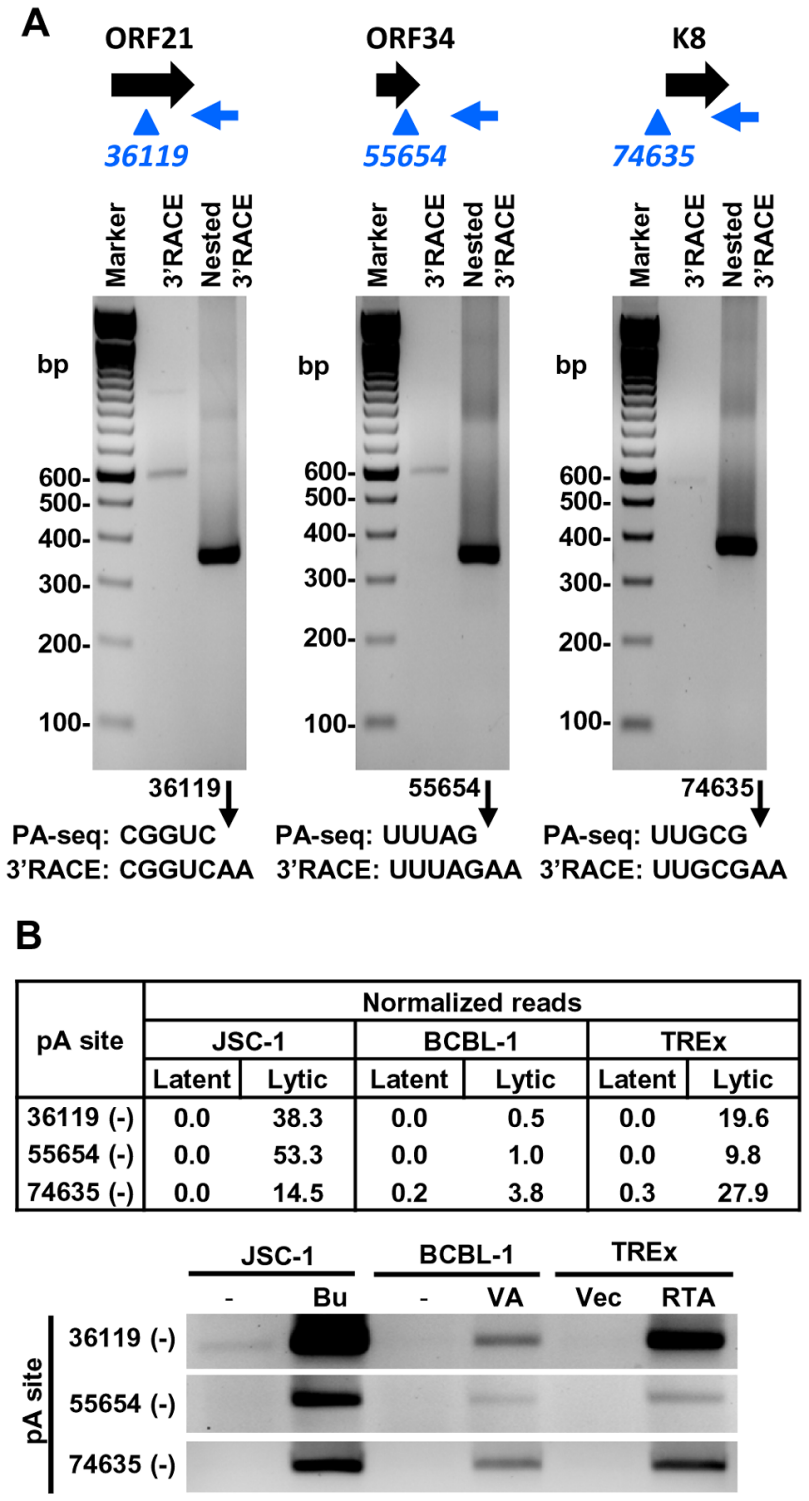
Validation of PA-seq-identified antisense RNAs to ORF21, ORF34, and ORF K8 by 3′ RACE. (A) 3′ RACE strategy, RACE product and sequencing result of the antisense RNA to ORF21, ORF34 or ORF K8. See Figure 6 for more details. (B) Detection of 3′ RACE products is correlated to the abundance of PA-seq reads derived from specific antisense RNAs in individual B cell lines with latent and lytic KSHV infection.

We further verified the PA-seq-identified pA sites from the mRNAs antisense to ORF21, ORF34, and ORF K8 by 3′ RACE and confirmed the production of the antisense RNAs in B cells during viral lytic infection (Figure 7A). The read abundance of these novel pA sites associated with each antisense RNA was correlated, as predicted, to the amount (measured by band intensity) of the 3′ RACE products derived from its corresponding RNA transcript (Figure 7B).

### Alternative polyadenylation of KSHV T1.5 RNA

**Figure 8:**
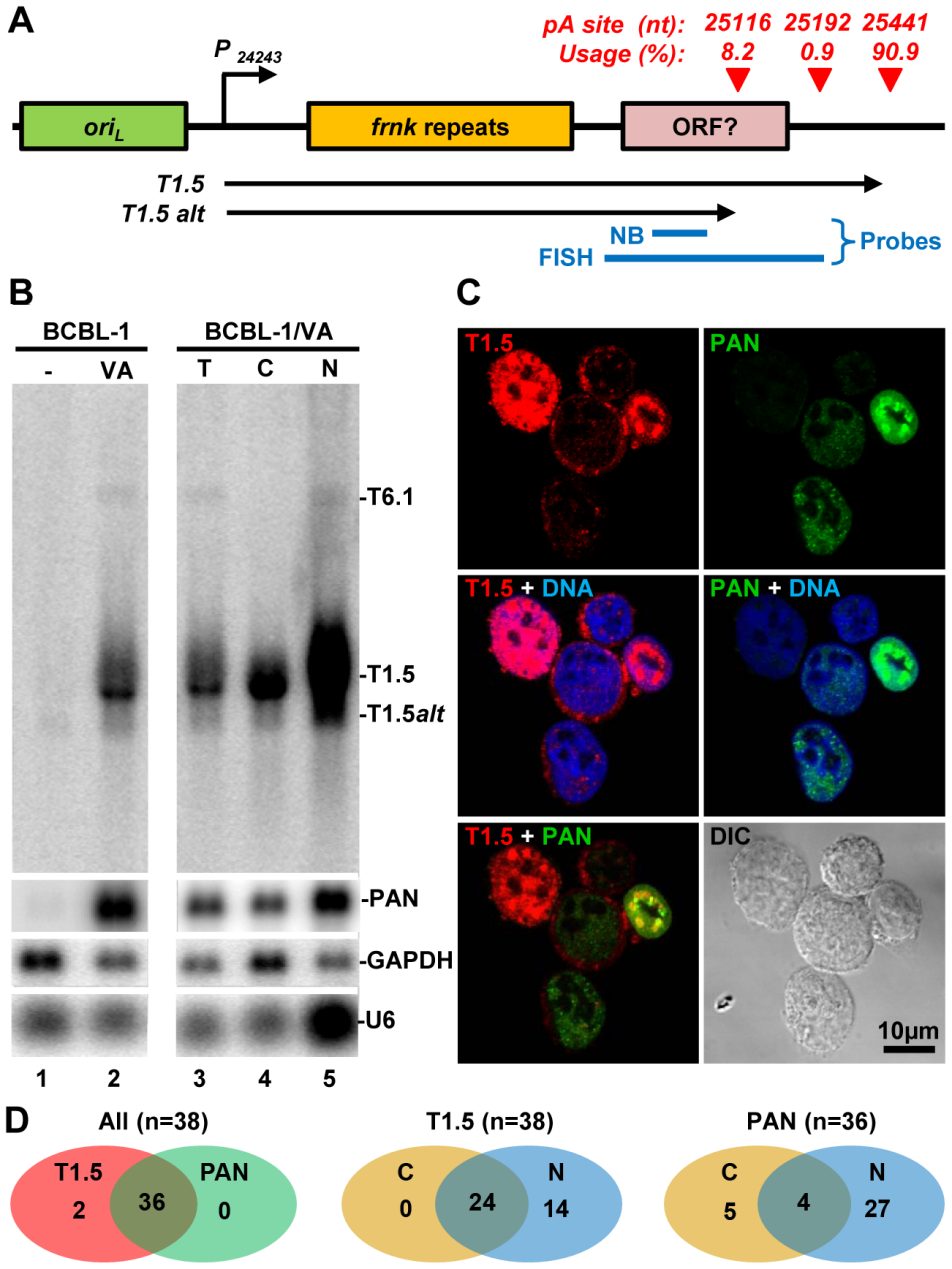
Subcellular localization of KSHV T1.5 lncRNA in PEL cells. (A) Diagram displaying the gene structure of T1.5 locus with a cluster of pA sites identified by PA-seq (red triangles), Blue lines represent probes used for Northern blot (NB) and RNA FISH. P –promoter, *ori_L_*-lytic origin of replication. (B) Northern blot analysis of total (T) or fractionated (C-cytoplasmic, N-nuclear) RNA isolated from BCBL-1 cells 24 h after induction with 1 mM sodium valproate (VA). A ^32^P-labeled antisense oligo specific for T1.5, PAN, GAPDH, or U6 was used as a probe. (C and D) RNA FISH assay was carried out in TREx BCBL1-RTA cells induced with 0.1 µg/ml of doxycyclin for 24 h. After induction the cells were fixed and hybridized with Alexaflour-labeled antisense RNA probes prepared by *in vitro* transcription from plasmids containing KSHV DNA fragments corresponding to T1.5 (red) or PAN (green) RNA. Cell nuclei were counterstained by Hoechst DNA dye. The subcellular distributions of T1.5 and PAN RNAs in TREx BCBL1-RTA cells were examined by confocal microscopy (C). The number of the B cells with coexpression and subcellular (C, cytoplasmic; N, nuclear) T1.5 and/or PAN RNAs are summarized in Venn diagrams (D).

KSHV T1.5 RNA is a long non-coding RNA, which is transcribed from nt 24243 in the KSHV genome, next to the left lytic origin of replication (*ori_L_*)(Figure 8A). The expression of T1.5 RNA is strongly inducible by viral transactivator RTA [32]. While the expression of T1.5 is required for viral DNA replication, its functional characteristics remain unknown [33]. Our study showed that T1.5 RNA is one of the most abundant transcript expressed during KSHV infection. The T1.5 RNA 3′ end was mapped to nt 25440 [34] and we mapped it to nt 25441 by PA-seq and by 3′RACE (Figure 6). In addition, we found that about 10% of T1.5 transcripts were also polyadenylated from two additional pA sites upstream of the nt 22541 pA site (Figure 8A), leading to the production of ∼300 nts shorter transcripts as verified by Northern blot analysis of BCBL-1 total RNA (Figure 8B). Because the antisense probe used in the assay was derived from a upstream region of the mapped pA sites, this probe could detect all transcripts running over this region: a strong band corresponding to the reported size of the inducible T1.5 RNA, a smaller size band (∼1.2 kb) with weaker intensity representing the alternatively polyadenylated T1.5 transcripts, and a much larger T6.1 transcript. The T6.1 RNA does not use T1.5 pA sites [34], but rather a PAN pA site for its expression (Figure 1A).

T1.5 RNA contains a few short ORFs and has potential to encode small peptides [34]. We thus assumed that T1.5 might be exportable to the cytoplasm. As expected, we demonstrated by Northern blot analysis its partial presence in the cytoplasm (Figure 8B). RNA FISH assays further showed T1.5 RNA distribution both in the cytoplasm and nucleus of KSHV infected PEL cells using an antisense RNA probe to the 3′ end of T1.5 (Figure 8A, Figure S5). In these two assays, nuclear PAN RNA served as a control (Figure 8B–C, Figure S5) and displayed, as expected, predominantly in the nucleus overlapping with Hoechst DNA staining [8], [35]. Interestingly, the nuclear coexpression of T1.5 and PAN RNA appears mutually exclusive. We found that the cells expressing high level of nuclear T1.5 RNA display much less nuclear PAN RNA or vice versa (Figure 8C). Compared with the subcellular distribution profile of PAN RNA, we saw more B cells with both cytoplasmic and nuclear distribution of T1.5 RNA during virus lytic infection (Figure 8D).

### Application of PA-seq to examine the expression of host IL6 and GAPDH RNA in B cells with KSHV lytic infection

**Figure 9:**
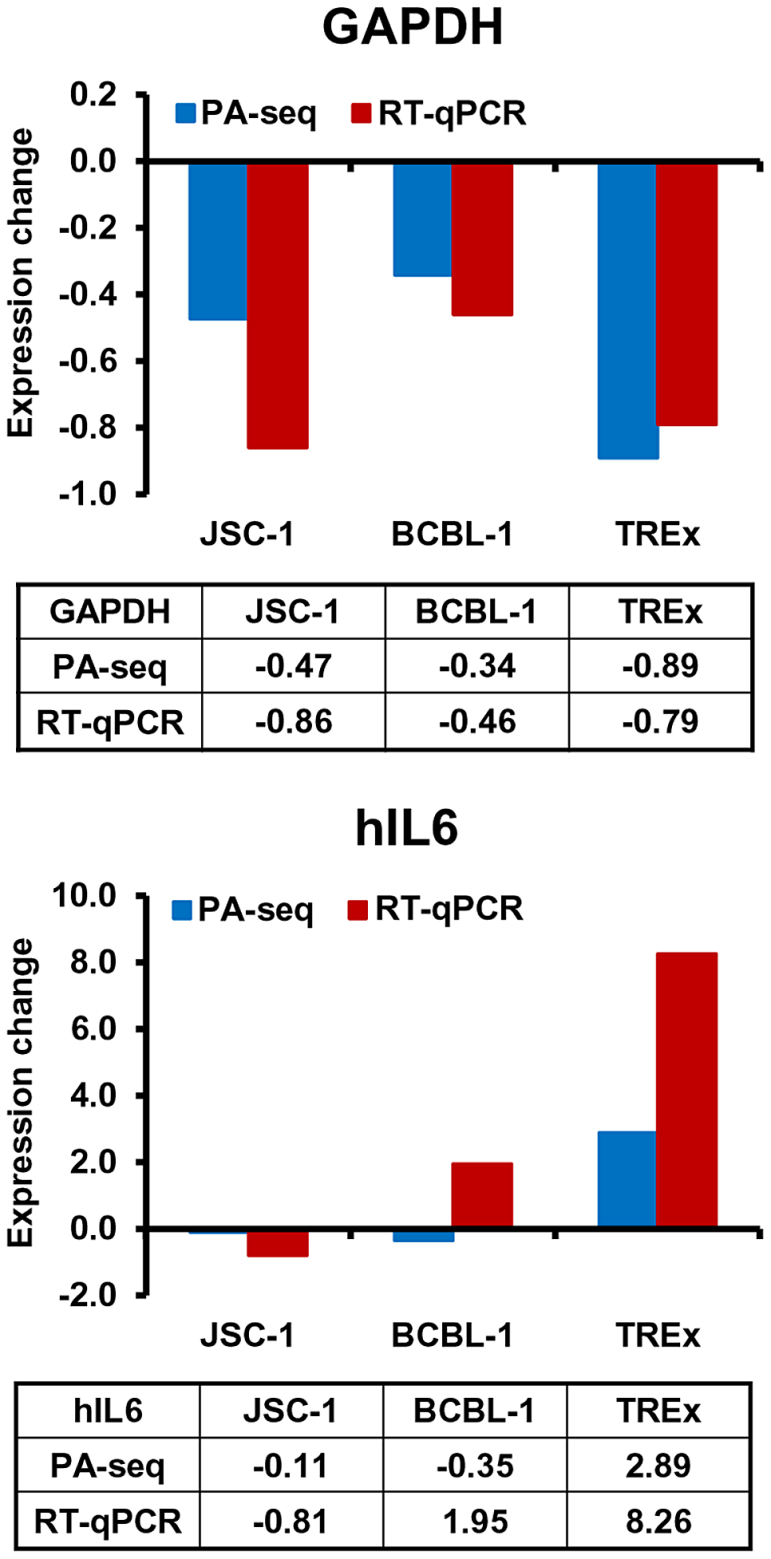
Application of PA-seq to examine the expression of host IL-6 and GAPDH during KSHV lytic infection. Bar graphs and tables below each bar graph show the quantitative RNA levels of GAPDH and human IL6 (hIL6).

The usefulness of PA-seq was further extended to examine the expression of a few host genes for its possible application to unveil a pA site landscape of the host genome before and after KSHV lytic infection. Human IL6 (hIL6) and GAPDH were initially chosen because B cells with lytic KSHV infection exhibit increased expression of human IL6 [36], [37], but decreased expression of GAPDH (Figure 8B). As shown in Figure 9, the results from PA-seq on GAPDH and hIL6 were comparable with that from RT-qPCR. The decreased expression of GAPDH RNA could be found by both methods in all three tested B cell lines with KHSV lytic infection and a significant increase of hIL6 expression in TREx BCBL-1 cells with lytic KSHV infection. However, we did not see in either method an increased hIL6 expression in JSC-1 cells with butyrate (a very potent inducer)-induced KSHV and EBV lytic coinfections, but observed the increased hIL6 expression in BCBL-1 cells with valproate (a weak inducer)-induced lytic KSHV infection by RT-qPCR. Human IL6 is a cytokine highly sensitive to (vulnerable for) RNA degradation and PA-seq detects the transcripts carrying an intact 3′-end poly (A) tail, while RT-qPCR detects only a small region of the IL6 RNA. Thus, multiple factors could contribute to the variations in detection of hIL6 gene expression from one cell line to another.

## Discussion

In this report we present the first viral genome landscape of polyadenylation sites from three PEL cell lines with KSHV latent or lytic infection. The comprehensive pA site landscape for the entire KSHV genome was revealed by using a modified PA-seq strategy which conveys single nucleotide resolution and strand specificity [24], [25]. The mapped pA sites have been annotated to all known KSHV genes and four putative novel genes in the KSHV genome. The steady-state expression level of every gene in the KSHV genome from viral latent to lytic infection was quantified by PA-seq reads associated with each mapped pA site and was used to distinguish viral latent genes from lytic genes. By analyzing the flanking sequences of each mapped pA site, we determined the regulatory elements governing viral RNA polyadenylation and gene expression. More importantly, we identified several viral genes utilizing alternative polyadenylation as a mechanism for their expression during KSHV infection. In general, the mapped viral pA sites in this study have high accuracy both in terms of nucleotide position and strand orientation, when compared with the known viral pA sites identified by the conventional methods (Table S3) [8], [34], [38]–[42]. However, we were unable to verify a few pA sites previously reported in other studies, including a pA site at nt 124061 for a C-terminal truncated LANA [43] and a pA site at nt 18200 (+) for the expression of a 0.7 kb transcript antisense to K2 (vIL-6) [29], [31]. The 0.7 kb transcript was discovered using custom-made tiling arrays covering the entire KSHV genome [29], [31] and a T7-Oligo(dT) primer for sample cDNA synthesis. The likelihood internal priming of the oligo primer used in the study might create aberrant synthesis of cDNA probes hybridizing to the tiling arrays. In our study the detection of any pseudo pA sites resulting from internal priming was largely avoided by exclusion of the sequence reads upstream of an A-stretch in the KSHV genome. It is worth noting that the expression of the 0.7 kb transcript antisense to K2 was originally discovered only in viral lytically-infected endothelial iSLK.219 cells derived from Kaposi sarcoma, but not detected in PEL-derived B cells [29]. In addition to assigning the known pA sites and many novel viral pA sites from this study to the KSHV genes being previously annotated, we also identified a few novel viral pA sites (Table S3) that could not be assigned to any known KSHV genes. These unassigned pA sites are often found in the opposite strand to known KSHV genes, including ORF8, ORF21, ORF34, K8 and ORF50. Some of those antisense transcripts were described in other reports [29] and the existence of these RNAs antisense to ORF21, ORF34, and ORF K8 transcripts could be confirmed by 3′ RACE in this study (Figure 7). Their potential roles in KSHV biology are now under active investigation.

KSHV has been evolved to use one pA site for the expression of multiple genes in many regions of the genome. Supporting this notion, our PA-seq analysis identified numerous regions of the KSHV genome with several viral genes (up to 5 genes) sharing a common pA site (Figure 1B). As a consequence, many KSHV genes are expressed as bicistronic or polycistronic transcripts with a long 3′ UTR covering the coding region (s) of downstream gene (s). These RNA structures are vulnerable to viral and cellular miRNAs [44]–[46] and all transcripts from the gene cluster regions could be regulated even by a single miRNA. Others could avoid this regulation by RNA splicing of the downstream ORF(s) as shown in ORF50/K8/K8.1 and K1 transcript [28], [47], [48]. Thus, understanding the gene organization and pA site position is critical for knocking-out or knocking-down studies of various virus genes from the KSHV genome in order to make appropriate interpretation on the function of individual viral genes in a cluster region.

The usage of each mapped pA site in this study was determined by counting the sequence reads associated with each pA site to approximate the steady-state expression level of the associated gene(s). When the sequence-reads of a given pA site in viral lytic infection were compared with that in viral latent infection, we could distinguish pA site usage from viral lytic genes to viral latent genes. The pA site for lytic gene expression could be used 100-fold more in lytic infection than in latent infection, whereas the pA site usage for latent gene expression displays only little increase (less than 10-folds) in lytic infection. Two pA sites downstream of K12, a classical viral latent gene, could be an exception because both showed an increased usage in viral lytic infection. The increased usage of two K12 pA sites is consistent with the finding that a lytic inducible promoter could be activated for K12 expression [49]. Analysis of pA site usage in lytic viral infection also confirmed PAN RNA being an extremely abundant RNA species, with sequence-read counts in the mapped pA site at nt 29740 (+) from viral lytic infection alone representing more than 80% of the total sequence-reads for all pA sites.

Moreover, the efficient expression of viral RNA transcripts was found being related to the peak size of a pA site in this study. It should note that each transcript could give rise to only one read in PA-seq. Thus, the read count in a PA peak simply reflects the abundance of the corresponding transcript. In fact, more sequence reads are not expected to inflate the size of a PA peak, especially when the pA cleavage events are precise. Therefore, the positive correlation we observed between the sizes of PA peaks and the expression levels of corresponding transcripts may suggest some degree of “slippage” in polyadenylation of viral transcripts to ensure high-level expression at the lytic stage. When compared to the pA sites falling into a broad or wide peak, an RNA transcript carrying a pA site with a narrow peak was less expressed, with fewer PA-seq sequence reads. Although this difference in the pA sites with a narrow peak might be attributable partially to their frequent usage of non-canonical PAS, there must be other unknown mechanisms governing the utilization of a pA site with a narrow peak, other than canonical vs non-canonical PAS *per se*. Previous reports showed that the PAS strength directly affects the overall level of mature transcripts [50], [51] and is determined by conservation of RNA cis-elements UGUAN upstream and an run of U/G downstream of the PAS AAUAAA. For example, the presence of a weaker early SV40 PAS leads to lower expression of a reporter gene than the construct containing a stronger SV40 late PAS when both were driven by the same promoter [52]. Therefore, the PAS strength governing polyadenylation of individual viral transcripts may provide additional level of regulation to fine tune their proper expression during viral infection. In addition, the length of the 3′ UTR could be another factor to affect RNA expression level. A shorter 3′ UTR in KSHV transcripts would provide expression advantage of viral genes in escaping from miRNA-mediated RNA degradation [53], [54].

Recent studies unveiled highly prevalent alternative RNA polyadenylation in various organisms and its profound role in regulation of gene expression [23]. We identified several KSHV genes, including both non-coding and protein-coding genes, exhibit alternative RNA polyadenylation (Table S4). These alternative pA sites were previously ignored because of their relatively lower prevalence and the conceptual bias toward the longest detectable transcripts. All alternative pA sites identified in our study were located in the 3′ UTR of the respective transcripts and thus, their utilization does not affect coding potential of these variant transcripts. Notably, alternative polyadenylation was identified in two most abundant viral lncRNAs PAN and T1.5, each of which harbors three alternative pA sites. We experimentally verified the two alternative pA sites for the expression of corresponding T1.5 transcripts in B cells with viral lytic infection (Figures 6, 8). In addition, alternative polyadenylation of PAN RNA expression had been reported in our earlier study [35]. Therefore, the role of alternative polyadenylation in PAN and T1.5 expression will become an attractive subject for better understanding the function of PAN and T1.5 lncRNAs.

Two unusual clusters of pA sites located downstream of the internal repeat regions were identified by PA-seq, but could not be validated by 3′ RACE in this study. The first cluster is located in the minus strand of the KSHV genome, downstream of “*vnct*” 13-bp repeats and composed of 5 individual pA sites within a ∼250-bp region from nt 29376 (-) to 29615 (-). The second cluster of three pA sites from 118012 (-) to 118087 (-) is also located in the minus strand downstream of “*zppa*” repeat region containing two 23-bp repeats (Figure 1A). These pA sites are located within the coding region of K12, but no transcripts associated with these mapped pA sites were detected in previous studies [40]. None of them has a canonical PAS upstream. The sequence reads detected by PA-seq are more likely associated with cryptic transcription from the internal regions [55]. However, these transcripts are unstable and their degradation by cellular exosome is initiated by addition of a short pA tail, which is mediated by a non-canonical pA polymerase and is therefore is not dependent on PAS [56]. These transcripts with the rapid turnover may not be detectable by 3′RACE, but could be picked up by our high sensitive PA-seq.

## Materials and Methods

### Cells

Primary effusion lymphoma cells lines (JSC-1 [KSHV+, EBV+], BCBL-1 [KSHV+ only] and TREx BCBL-1-vector and –RTA [BCBL-1 derived]) [57], [58] were used in this study The viral lytic replication was induced for 48 h by 3 mM sodium butyrate (Bu) for JSC-1 cells, 0.6 mM sodium valproate (VA) for BCBL-1 cells, or 1 µg/ml doxycycline (DOX) for both TREx BCBL-1-vector and –RTA cells. Total RNA was isolated by TRIzol (Invitrogen) and genomic DNA contamination was removed by RNeasy Mini kit (Qiagen) using on-column DNase I digestion step.

### PA-seq

The 3′end library for each sample was constructed using a modified PA-seq strategy [25], [24]. Briefly, 10 µg of DNA-free total RNA from each sample described above was sheared into 200–300 nt fragments by heating (94°C for 3 minutes) with magnesium. After precipitation a reverse transcription was carried out using a modified oligo(dT) primer (5′-bio-T_16_dUTTTVN-3′, ‘bio’ denotes duo biotin group, ‘dU’ stands for deoxyuridine, ‘V’ represents any nucleotide except T and ‘N’ denotes any nucleotide). After second strand synthesis, resulted dsDNA was pulled down by Dynabeads MyOne C1 (Invitrogen) and dephosphorylated with APex Heat-Labile Alkaline Phosphatase (Epicentre) enabling PCR strand specificity for selective adaptor ligation. Dephosphorylated dsDNA was released from beads by USER enzyme digestion (NEB) and end-repaired, followed by an “A” base addition at the ends. Notably, only the first-stand cDNA contains a 5′ phosphate, and thus can be ligated to bar-coded *Illumina* paired-end Y linker without a nick. The usage of a dUTP in the oligo(dT) primer and the de-phosphorylation step reinforce strand-specificity, and allow precisely mapping of pA cleavage site at singe-base resolution. Ligation products between 250 bp and 450 bp were gel purified and PA-seq libraries were generated by 16-cycle PCR with Phusion Hot Start High-Fidelity DNA Polymerase (Finnzymes). The obtained libraries were subjected to two technical replicate sequencing by an *Illumina* HiSeq2000 sequencer.

### Sequence analysis

Obtained raw reads were first aligned to KSHV genome (GenBank acc no U75698.1), EBV B95-8 strain genome (GenBank acc no V01555.2) and human genome (UCSC version hg19) by Burrows-Wheeler Alignment tool (BWA) [59] allowing two mismatches and processed by SAMtools [60]. All uniquely mapped KSHV-specific sequence pairs were used for downstream analyses. First the distribution of obtained reads along KSHV genome was visualized using IGV genome browser (www.broadinstitute.org/igv/) to assure their suitability for pA site analysis. Individual KSHV pA sites were then designated by peak calling using F-Seq program [26] on combined libraries. The PA-seq peaks above the threshold of 50 reads were considered as true peaks. The peaks were further refined by removing pseudo pA sites resulting from “internal priming” due to continuous “A-stretch” in the template. After the peak calling the sequence reads were assigned back to individual samples to obtain the reads-counts for both latent and lytic infection. To obtain a relative expression level the total reads-counts were normalized per million to overall reads mapped to both KSHV and human [61].

### Sequence motifs analysis

The sequence surrounding the mapped pA sites was covered from 50 nts upstream and 50 nts downstream of each identified pA site for the motif analysis. The percentage of occurrence for each nucleotide was calculated, plotted and smoothed with the loess function in R software (R version 2.12.1). Polyadenylation signals (PAS) occurred within 50 nts upstream of pA site were assigned manually. Graphical representation of sequence conservation was generated by Weblogo v3 (http://weblogo.berkeley.edu/) [62], [63].

### 3′ RACE

Transcript 3′ end was identified by SMARTer RACE cDNA Amplification Kit (Clontech). The primer sequences used in 3′RACE are listed in Table S11. The obtained 3′RACE products were sequenced directly or after cloning in pCR2.1-TOPO vector (Invitrogen).

### Northern blot

Total RNA was isolated using TRIzol reagent. The cytoplasmic and nuclear fractions of RNA were isolated as described [64]. Obtained RNA (5 µg) was separated on agarose gel and analyzed by Northern blot analysis with ^32^P labeled oligo probes: oVM 208 (5′-CGTGGCTGTGCTTCTCATCAT-3′) for T1.5 lncRNA, oJM7 (5′-GTTACACAACGCTTTCACCTACA-3′) for PAN lncRNA, oZMZ270 (5′-TGAGTCCTTCCACGATACCAAA-3′) for GAPDH and oST197 (5′-AAAATATGGAACGCTTCACGA-3′) for U6 snRNA.

### RNA FISH

The single stranded sense and antisense RNA probes were prepared by FISH Tag RNA Multicolour Kit (Invitrogen) by *in vitro* transcription using DNA fragment of KSHV genome (nt 24906–25375 for T1.5 and nt 29018–29481 for PAN lncRNAs) as templates. The hybridization was performed as previously described [65]. After immobilization the cells were fixed with 2% paraformaldehyde, permeabilized with 0.5% Triton X-100 and blocked with hybridization buffer (50% formamid, 5×SSC, 0.1% Tween-20, 50 µg/ml heparin, 100 µg/ml salmon DNA). The hybridization was carried out overnight at 55°C. The nuclei were counterstained with Hoechst dye. The pictures were collected using a Zeiss LSM510 META laser-scanning microscope (Zeiss).

### RT-qPCR

Total cell RNA isolated by TRIzol (Invitrogen) was treated with Turbo DNA-free DNase to remove DNA. Five micrograms of total cell RNA was used to synthesize cDNA using SuperScript First-Stand Synthesis System (Invitrogen). The GAPDH and human IL6 (hIL6) transcript levels were determined by RT-qPCR using *ΔC_t_* method [44], [66], [67].

## Supporting Information

Figure S1 **PA-seq analysis of KSHV transcripts.** (A) Three KSHV-infected PEL (primary effusion lymphoma)-derived B-cell lines were used in PA-seq analysis during virus latent infection (left column) or lytic infection (right column). (B) Total numbers of sequence reads from each sample mapped to KSHV genome (Genbank acc no U75698.1) or human genome (UCSC version hg19). The other unassigned reads including those mapped to EBV genome (Genbank acc no V01555.2) (3233 reads or 0.02% in latent and 1998744 reads or 13.11% in lytic infection of JSC-1 cells) are shown as others. (C) A bar graph depicting % distribution of the sequence reads from each sample assigned to KSHV or human genome or others unassigned reads.
(TIF)

Figure S2 **Determination of KSHV pA sites by F-seq analysis.** Diagram shows PA peak (red line) identified by F-seq analysis of viral sequence reads (blue bars) aligned to the KSHV genome. The PA mode, a nucleotide position with the highest number of reads within the peak, was designated as a pA site. The peak size is a distance from nucleotide position of the beginning to the end of the peak within which a pA site is assigned. The total number of all reads within the peak represents usage of the pA site.
(TIF)

Figure S3 **Visual distribution of KSHV-specific sequence reads obtained by PA-seq across viral genome.** (A) Positions and frequency (scaled to maximal 500) of the sequence reads derived from B cells with latent (blue bars) or lytic (red bars) infection were visualized on KSHV genome by IGV software (http://www.broadinstitute.org/igv/). Green lines in the middle represent positions of reported KSHV genes. (B) A zoom-in to the locus containing ORF50 (RTA)-K8-K8.1 gene cluster where the sequence reads distribute in a plus (+) strand of the KSHV genome. Below is a diagram of previously reported gene structure and primary transcripts associated with this gene locus. Boxes represent an ORF with positions of mapped promoters (arrows) and a pA cleavage site (CS). The reads in latent infection represent spontaneous reactivation of this locus in a very small fraction of BCBL-1 cells and BCBL-1-derived TREx cells.
(TIF)

Figure S4 **Illustration of pA site mapped to the KSHV genome in individual B cell lines with latent (blue) or lytic (red) KSHV infection.** Scaled bars for each pA site represent normalized PA-seq reads per million.
(TIF)

Figure S5 **Localization of KSHV T1.5 and PAN lncRNAs in PEL cells.** Specificity of each probe described in Figure 8 was tested in doxycycline-treated TREx cells by RNA FISH experiment as described in experimental procedures. The specific signal was observed only in TREx-RTA cells but not in TREx-vector cells.
(TIF)

Table S1 **Positions and strand specificity of all KSHV pA sites determined by F-seq analysis of combined six PA-seq libraries.**
(PDF)

Table S2 **The pA sites mapped by PA-seq in selected KSHV viral transcripts are comparable to the pA sites previously mapped by traditional methods.**
(PDF)

Table S3 **Utilization of identified pA site with individual or cluster of KSHV genes.** Adjacent 3′UTR length calculated as a distance between mapped KSHV sites to an immediately upstream KSHV ORF. N/A-not applicable.
(PDF)

Table S4 **KSHV genes contain alternative pA sites which can be used during virus infection.** Individual pA site usage (%) was calculated from total number of sequence reads for all pA sites in a given gene transcript.
(PDF)

Table S5 **Normalized pA site reads mapped to the KSHV genome in individual B cell lines with latent or lytic KSHV infection.**
(PDF)

Table S6 **The usage of individual KSHV pA sites during latent and lytic infection from combined datasets of three PEL cell lines (Table S5).** The fold increase for each pA site was calculated by dividing the sequence reads from lytic samples by number of the reads from latency. N/A-not applicable.
(PDF)

Table S7 **Classification of KSHV pA sites based on the PA peak size.** The size of each peak was calculated as a distance between start and end of the peak and it was correlated with pA site usage (Table S1). Based on their peak size, all pA sites were divided into three categories: narrow (NP, ≤30 nts), broad (BP, >30, ≤45 nts) or wide (WP, >45 nts) peaks.
(PDF)

Table S8 **Frequency of pA site usage in correlation to PA peak size.** The Pearson (*r*) and Spearman (*r_s_*) correlation coefficients expressing correlation between peak size and number of reads were calculated for each group of pA site.
(PDF)

Table S9 **Canonical and non-canonical PAS (red) detected in a region covering 50 nts immediately upstream of the mapped pA site.** N/D-not detectable.
(PDF)

Table S10 **Prevalence of canonical and non-canonical PAS 50 nts upstream of the mapped pA sites in all, narrow, broad, wide, top 10, and bottom 10 pA sites.**
(PDF)

Table S11 **Primers used in 3′RACE analyses.**
(PDF)

## References

[1] Russo JJ, Bohenzky RA, Chien MC, Chen J, Yan M, et al (1996) Nucleotide sequence of the Kaposi sarcoma-associated herpesvirus (HHV8). Proc Natl Acad Sci U S A 93: 14862–14867. 8962146 10.1073/pnas.93.25.14862 PMC26227

[2] Chang Y, Cesarman E, Pessin MS, Lee F, Culpepper J, et al (1994) Identification of herpesvirus-like DNA sequences in AIDS-associated Kaposi's sarcoma. Science 266: 1865–1869. 7997879 10.1126/science.7997879

[3] Cesarman E, Chang Y, Moore PS, Said JW, Knowles DM (1995) Kaposi's sarcoma-associated herpesvirus-like DNA sequences in AIDS-related body-cavity-based lymphomas. N Engl J Med 332: 1186–1191. 7700311 10.1056/NEJM199505043321802

[4] Dupin N, Diss TL, Kellam P, Tulliez M, Du MQ, et al (2000) HHV-8 is associated with a plasmablastic variant of Castleman disease that is linked to HHV-8-positive plasmablastic lymphoma. Blood 95: 1406–1412. 10666218

[5] Sun R, Lin SF, Gradoville L, Yuan Y, Zhu F, et al (1998) A viral gene that activates lytic cycle expression of Kaposi's sarcoma-associated herpesvirus. Proc Natl Acad Sci U S A 95: 10866–10871. 9724796 10.1073/pnas.95.18.10866 PMC27987

[6] Sun R, Lin SF, Staskus K, Gradoville L, Grogan E, et al (1999) Kinetics of Kaposi's sarcoma-associated herpesvirus gene expression. J Virol 73: 2232–2242. 9971806 10.1128/jvi.73.3.2232-2242.1999 PMC104468

[7] Lukac DM, Kirshner JR, Ganem D (1999) Transcriptional activation by the product of open reading frame 50 of Kaposi's sarcoma-associated herpesvirus is required for lytic viral reactivation in B cells. J Virol 73: 9348–9361. 10516043 10.1128/jvi.73.11.9348-9361.1999 PMC112969

[8] Sun R, Lin SF, Gradoville L, Miller G (1996) Polyadenylylated nuclear RNA encoded by Kaposi sarcoma-associated herpesvirus. Proc Natl Acad Sci U S A 93: 11883–11888. 8876232 10.1073/pnas.93.21.11883 PMC38153

[9] Cai X, Lu S, Zhang Z, Gonzalez CM, Damania B, et al (2005) Kaposi's sarcoma-associated herpesvirus expresses an array of viral microRNAs in latently infected cells. Proc Natl Acad Sci U S A 102: 5570–5575. 15800047 10.1073/pnas.0408192102 PMC556237

[10] Xu Y, Ganem D (2010) Making sense of antisense: seemingly noncoding RNAs antisense to the master regulator of Kaposi's sarcoma-associated herpesvirus lytic replication do not regulate that transcript but serve as mRNAs encoding small peptides. J Virol 84: 5465–5475. 20357088 10.1128/JVI.02705-09 PMC2876621

[11] Colgan DF, Manley JL (1997) Mechanism and regulation of mRNA polyadenylation. Genes Dev 11: 2755–2766. 9353246 10.1101/gad.11.21.2755

[12] Lewis JD, Gunderson SI, Mattaj IW (1995) The influence of 5′ and 3′ end structures on pre-mRNA metabolism. J Cell Sci Suppl 19: 13–19. 10.1242/jcs.1995.supplement_19.2 8655642

[13] Wickens M, Anderson P, Jackson RJ (1997) Life and death in the cytoplasm: messages from the 3′ end. Curr Opin Genet Dev 7: 220–232. 9115434 10.1016/s0959-437x(97)80132-3

[14] Shi Y, Di G, Taylor D, Sarkeshik A, Rice WJ, et al (2009) Molecular architecture of the human pre-mRNA 3′ processing complex. Mol Cell 33: 365–376. 19217410 10.1016/j.molcel.2008.12.028 PMC2946185

[15] Salisbury J, Hutchison KW, Graber JH (2006) A multispecies comparison of the metazoan 3′-processing downstream elements and the CstF-64 RNA recognition motif. BMC Genomics 7: 55. 16542450 10.1186/1471-2164-7-55 PMC1539018

[16] Hu J, Lutz CS, Wilusz J, Tian B (2005) Bioinformatic identification of candidate cis-regulatory elements involved in human mRNA polyadenylation. RNA 11: 1485–1493. 16131587 10.1261/rna.2107305 PMC1370832

[17] Bagga PS, Ford LP, Chen F, Wilusz J (1995) The G-rich auxiliary downstream element has distinct sequence and position requirements and mediates efficient 3′ end pre-mRNA processing through a trans-acting factor. Nucleic Acids Res 23: 1625–1631. 7784220 10.1093/nar/23.9.1625 PMC306907

[18] Chen F, Wilusz J (1998) Auxiliary downstream elements are required for efficient polyadenylation of mammalian pre-mRNAs. Nucleic Acids Res 26: 2891–2898. 9611233 10.1093/nar/26.12.2891 PMC147640

[19] Millevoi S, Vagner S (2010) Molecular mechanisms of eukaryotic pre-mRNA 3′ end processing regulation. Nucleic Acids Res 38: 2757–2774. 20044349 10.1093/nar/gkp1176 PMC2874999

[20] Tian B, Hu J, Zhang H, Lutz CS (2005) A large-scale analysis of mRNA polyadenylation of human and mouse genes. Nucleic Acids Res 33: 201–212. 15647503 10.1093/nar/gki158 PMC546146

[21] Mangone M, Manoharan AP, Thierry-Mieg D, Thierry-Mieg J, Han T, et al (2010) The landscape of C. elegans 3′UTRs. Science 329: 432–435. 20522740 10.1126/science.1191244 PMC3142571

[22] Nagalakshmi U, Wang Z, Waern K, Shou C, Raha D, et al (2008) The transcriptional landscape of the yeast genome defined by RNA sequencing. Science 320: 1344–1349. 18451266 10.1126/science.1158441 PMC2951732

[23] Di G, Nishida K, Manley JL (2011) Mechanisms and consequences of alternative polyadenylation. Mol Cell 43: 853–866. 21925375 10.1016/j.molcel.2011.08.017 PMC3194005

[24] Ni T, Yang Y, Hafez D, Yang W, Kiesewetter K, et al (2013) Distinct polyadenylation landscapes of diverse human tissues revealed by a modified PA-seq strategy. BMC Genomics 14: 615. 24025092 10.1186/1471-2164-14-615 PMC3848854

[25] Hafez D, Ni T, Mukherjee S, Zhu J, Ohler U (2013) Genome-wide identification and predictive modeling of tissue-specific alternative polyadenylation. Bioinformatics 29: i108–i116. 23812974 10.1093/bioinformatics/btt233 PMC3694680

[26] Boyle AP, Guinney J, Crawford GE, Furey TS (2008) F-Seq: a feature density estimator for high-throughput sequence tags. Bioinformatics 24: 2537–2538. 18784119 10.1093/bioinformatics/btn480 PMC2732284

[27] Zhu FX, Cusano T, Yuan Y (1999) Identification of the immediate-early transcripts of Kaposi's sarcoma-associated herpesvirus. J Virol 73: 5556–5567. 10364304 10.1128/jvi.73.7.5556-5567.1999 PMC112613

[28] Tang S, Zheng ZM (2002) Kaposi's sarcoma-associated herpesvirus K8 exon 3 contains three 5′-splice sites and harbors a K8.1 transcription start site. J Biol Chem 277: 14547–14556. 11832484 10.1074/jbc.M111308200

[29] Chandriani S, Xu Y, Ganem D (2010) The lytic transcriptome of Kaposi's sarcoma-associated herpesvirus reveals extensive transcription of noncoding regions, including regions antisense to important genes. J Virol 84: 7934–7942. 20534856 10.1128/JVI.00645-10 PMC2916530

[30] Pauws E, van Kampen AH, van de Graaf SA, de Vijlder JJ, Ris-Stalpers C (2001) Heterogeneity in polyadenylation cleavage sites in mammalian mRNA sequences: implications for SAGE analysis. Nucleic Acids Res 29: 1690–1694. 11292841 10.1093/nar/29.8.1690 PMC31324

[31] Dresang LR, Teuton JR, Feng H, Jacobs JM, Camp DG, et al (2011) Coupled transcriptome and proteome analysis of human lymphotropic tumor viruses: insights on the detection and discovery of viral genes. BMC Genomics 12: 625. 22185355 10.1186/1471-2164-12-625 PMC3282826

[32] Wang Y, Li H, Chan MY, Zhu FX, Lukac DM, et al (2004) Kaposi's sarcoma-associated herpesvirus ori-Lyt-dependent DNA replication: cis-acting requirements for replication and ori-Lyt-associated RNA transcription. J Virol 78: 8615–8629. 15280471 10.1128/JVI.78.16.8615-8629.2004 PMC479094

[33] Wang Y, Tang Q, Maul GG, Yuan Y (2006) Kaposi's sarcoma-associated herpesvirus ori-Lyt-dependent DNA replication: dual role of replication and transcription activator. J Virol 80: 12171–12186. 17020951 10.1128/JVI.00990-06 PMC1676287

[34] Taylor JL, Bennett HN, Snyder BA, Moore PS, Chang Y (2005) Transcriptional analysis of latent and inducible Kaposi's sarcoma-associated herpesvirus transcripts in the K4 to K7 region. J Virol 79: 15099–15106. 16306581 10.1128/JVI.79.24.15099-15106.2005 PMC1315995

[35] Massimelli MJ, Majerciak V, Kruhlak M, Zheng ZM (2013) Interplay between polyadenylate-binding protein 1 and Kaposi's sarcoma-associated herpesvirus ORF57 in accumulation of polyadenylated nuclear RNA, a viral long noncoding RNA. J Virol 87: 243–256. 23077296 10.1128/JVI.01693-12 PMC3536381

[36] Aoki Y, Yarchoan R, Braun J, Iwamoto A, Tosato G (2000) Viral and cellular cytokines in AIDS-related malignant lymphomatous effusions. Blood 96: 1599–1601. 10942415

[37] Kang JG, Pripuzova N, Majerciak V, Kruhlak M, Le SY, et al (2011) Kaposi's Sarcoma-Associated Herpesvirus ORF57 Promotes Escape of Viral and Human Interleukin-6 from MicroRNA-Mediated Suppression. J Virol 85: 2620–2630. 21209110 10.1128/JVI.02144-10 PMC3067933

[38] Wang SS, Chang PJ, Chen LW, Chen LY, Hung CH, et al (2012) Positive and negative regulation in the promoter of the ORF46 gene of Kaposi's sarcoma-associated herpesvirus. Virus Res 165: 157–169. 22366521 10.1016/j.virusres.2012.02.010

[39] Majerciak V, Yamanegi K, Zheng ZM (2006) Gene structure and expression of Kaposi's sarcoma-associated herpesvirus ORF56, ORF57, ORF58, and ORF59. J Virol 80: 11968–11981. 17020939 10.1128/JVI.01394-06 PMC1676266

[40] Li H, Komatsu T, Dezube BJ, Kaye KM (2002) The Kaposi's sarcoma-associated herpesvirus K12 transcript from a primary effusion lymphoma contains complex repeat elements, is spliced, and initiates from a novel promoter. J Virol 76: 11880–11888. 12414930 10.1128/JVI.76.23.11880-11888.2002 PMC136876

[41] Sarid R, Wiezorek JS, Moore PS, Chang Y (1999) Characterization and cell cycle regulation of the major Kaposi's sarcoma-associated herpesvirus (human herpesvirus 8) latent genes and their promoter. J Virol 73: 1438–1446. 9882349 10.1128/jvi.73.2.1438-1446.1999 PMC103968

[42] Chiou CJ, Poole LJ, Kim PS, Ciufo DM, Cannon JS, et al (2002) Patterns of gene expression and a transactivation function exhibited by the vGCR (ORF74) chemokine receptor protein of Kaposi's sarcoma-associated herpesvirus. J Virol 76: 3421–3439. 11884567 10.1128/JVI.76.7.3421-3439.2002 PMC136009

[43] Canham M, Talbot SJ (2004) A naturally occurring C-terminal truncated isoform of the latent nuclear antigen of Kaposi's sarcoma-associated herpesvirus does not associate with viral episomal DNA. J Gen Virol 85: 1363–1369. 15166417 10.1099/vir.0.79802-0

[44] Kang JG, Majerciak V, Uldrick TS, Wang X, Kruhlak M, et al (2011) Kaposi's sarcoma-associated herpesviral IL-6 and human IL-6 open reading frames contain miRNA binding sites and are subject to cellular miRNA regulation. J Pathol 225: 378–389. 21984125 10.1002/path.2962 PMC3528401

[45] Lin HR, Ganem D (2011) Viral microRNA target allows insight into the role of translation in governing microRNA target accessibility. Proc Natl Acad Sci U S A 108: 5148–5153. 21402938 10.1073/pnas.1102033108 PMC3069182

[46] Bellare P, Ganem D (2009) Regulation of KSHV lytic switch protein expression by a virus-encoded microRNA: an evolutionary adaptation that fine-tunes lytic reactivation. Cell Host Microbe 6: 570–575. 20006845 10.1016/j.chom.2009.11.008 PMC2822622

[47] Chandriani S, Ganem D (2010) Array-based transcript profiling and limiting-dilution reverse transcription-PCR analysis identify additional latent genes in Kaposi's sarcoma-associated herpesvirus. J Virol 84: 5565–5573. 20219929 10.1128/JVI.02723-09 PMC2876603

[48] Pearce M, Matsumura S, Wilson AC (2005) Transcripts encoding K12, v-FLIP, v-cyclin, and the microRNA cluster of Kaposi's sarcoma-associated herpesvirus originate from a common promoter. J Virol 79: 14457–14464. 16254382 10.1128/JVI.79.22.14457-14464.2005 PMC1280212

[49] Matsumura S, Fujita Y, Gomez E, Tanese N, Wilson AC (2005) Activation of the Kaposi's sarcoma-associated herpesvirus major latency locus by the lytic switch protein RTA (ORF50). J Virol 79: 8493–8505. 15956592 10.1128/JVI.79.13.8493-8505.2005 PMC1143749

[50] Chao LC, Jamil A, Kim SJ, Huang L, Martinson HG (1999) Assembly of the cleavage and polyadenylation apparatus requires about 10 seconds in vivo and is faster for strong than for weak poly(A) sites. Mol Cell Biol 19: 5588–5600. 10409748 10.1128/mcb.19.8.5588 PMC84411

[51] West S, Proudfoot NJ (2009) Transcriptional termination enhances protein expression in human cells. Mol Cell 33: 354–364. 19217409 10.1016/j.molcel.2009.01.008 PMC2706331

[52] Carswell S, Alwine JC (1989) Efficiency of utilization of the simian virus 40 late polyadenylation site: effects of upstream sequences. Mol Cell Biol 9: 4248–4258. 2573828 10.1128/mcb.9.10.4248-4258.1989 PMC362504

[53] Mayr C, Bartel DP (2009) Widespread shortening of 3′UTRs by alternative cleavage and polyadenylation activates oncogenes in cancer cells. Cell 138: 673–684. 19703394 10.1016/j.cell.2009.06.016 PMC2819821

[54] Hausser J, Syed AP, Bilen B, Zavolan M (2013) Analysis of CDS-located miRNA target sites suggests that they can effectively inhibit translation. Genome Res 23: 604–615. 23335364 10.1101/gr.139758.112 PMC3613578

[55] Usdin K (2008) The biological effects of simple tandem repeats: lessons from the repeat expansion diseases. Genome Res 18: 1011–1019. 18593815 10.1101/gr.070409.107 PMC3960014

[56] Wyers F, Rougemaille M, Badis G, Rousselle JC, Dufour ME, et al (2005) Cryptic pol II transcripts are degraded by a nuclear quality control pathway involving a new poly(A) polymerase. Cell 121: 725–737. 15935759 10.1016/j.cell.2005.04.030

[57] Cannon JS, Ciufo D, Hawkins AL, Griffin CA, Borowitz MJ, et al (2000) A new primary effusion lymphoma-derived cell line yields a highly infectious Kaposi's sarcoma herpesvirus-containing supernatant. J Virol 74: 10187–10193. 11024147 10.1128/jvi.74.21.10187-10193.2000 PMC102057

[58] Nakamura H, Lu M, Gwack Y, Souvlis J, Zeichner SL, et al (2003) Global changes in Kaposi's sarcoma-associated virus gene expression patterns following expression of a tetracycline-inducible Rta transactivator. J Virol 77: 4205–4220. 12634378 10.1128/JVI.77.7.4205-4220.2003 PMC150665

[59] Li H, Durbin R (2009) Fast and accurate short read alignment with Burrows-Wheeler transform. Bioinformatics 25: 1754–1760. 19451168 10.1093/bioinformatics/btp324 PMC2705234

[60] Li H, Handsaker B, Wysoker A, Fennell T, Ruan J, et al (2009) The Sequence Alignment/Map format and SAMtools. Bioinformatics 25: 2078–2079. 19505943 10.1093/bioinformatics/btp352 PMC2723002

[61] Mortazavi A, Williams BA, McCue K, Schaeffer L, Wold B (2008) Mapping and quantifying mammalian transcriptomes by RNA-Seq. Nat Methods 5: 621–628. 18516045 10.1038/nmeth.1226 PMC13303166

[62] Schneider TD, Stephens RM (1990) Sequence logos: a new way to display consensus sequences. Nucleic Acids Res 18: 6097–6100. 2172928 10.1093/nar/18.20.6097 PMC332411

[63] Crooks GE, Hon G, Chandonia JM, Brenner SE (2004) WebLogo: a sequence logo generator. Genome Res 14: 1188–1190. 15173120 10.1101/gr.849004 PMC419797

[64] Majerciak V, Yamanegi K, Nie SH, Zheng ZM (2006) Structural and functional analyses of Kaposi sarcoma-associated herpesvirus ORF57 nuclear localization signals in living cells. J Biol Chem 281: 28365–28378. 16829516 10.1074/jbc.M603095200

[65] Pilkington GR, Majerciak V, Bear J, Uranishi H, Zheng ZM, et al (2012) Kaposi's sarcoma-associated herpesvirus ORF57 is not a bona fide export factor. J Virol 86: 13089–13094. 22993146 10.1128/JVI.00606-12 PMC3497679

[66] Massimelli MJ, Kang JG, Majerciak V, Le SY, Liewehr DJ, et al (2011) Stability of a Long Noncoding Viral RNA Depends on a 9-nt Core Element at the RNA 5′ End to Interact with Viral ORF57 and Cellular PABPC1. Int J Biol Sci 7: 1145–1160. 22043172 10.7150/ijbs.7.1145 PMC3204405

[67] Livak KJ, Schmittgen TD (2001) Analysis of relative gene expression data using real-time quantitative PCR and the 2(-Delta Delta C(T)) Method. Methods 25: 402–408. 11846609 10.1006/meth.2001.1262

