# Supplementary figures and images for: A Viral Genome Landscape of RNA Polyadenylation from KSHV Latent to Lytic Infection
Source: PLoS Pathog. 2013 Nov 14;9(11):e1003749. doi: 10.1371/journal.ppat.1003749 (PMC3828183; doi:10.1371/journal.ppat.1003749)

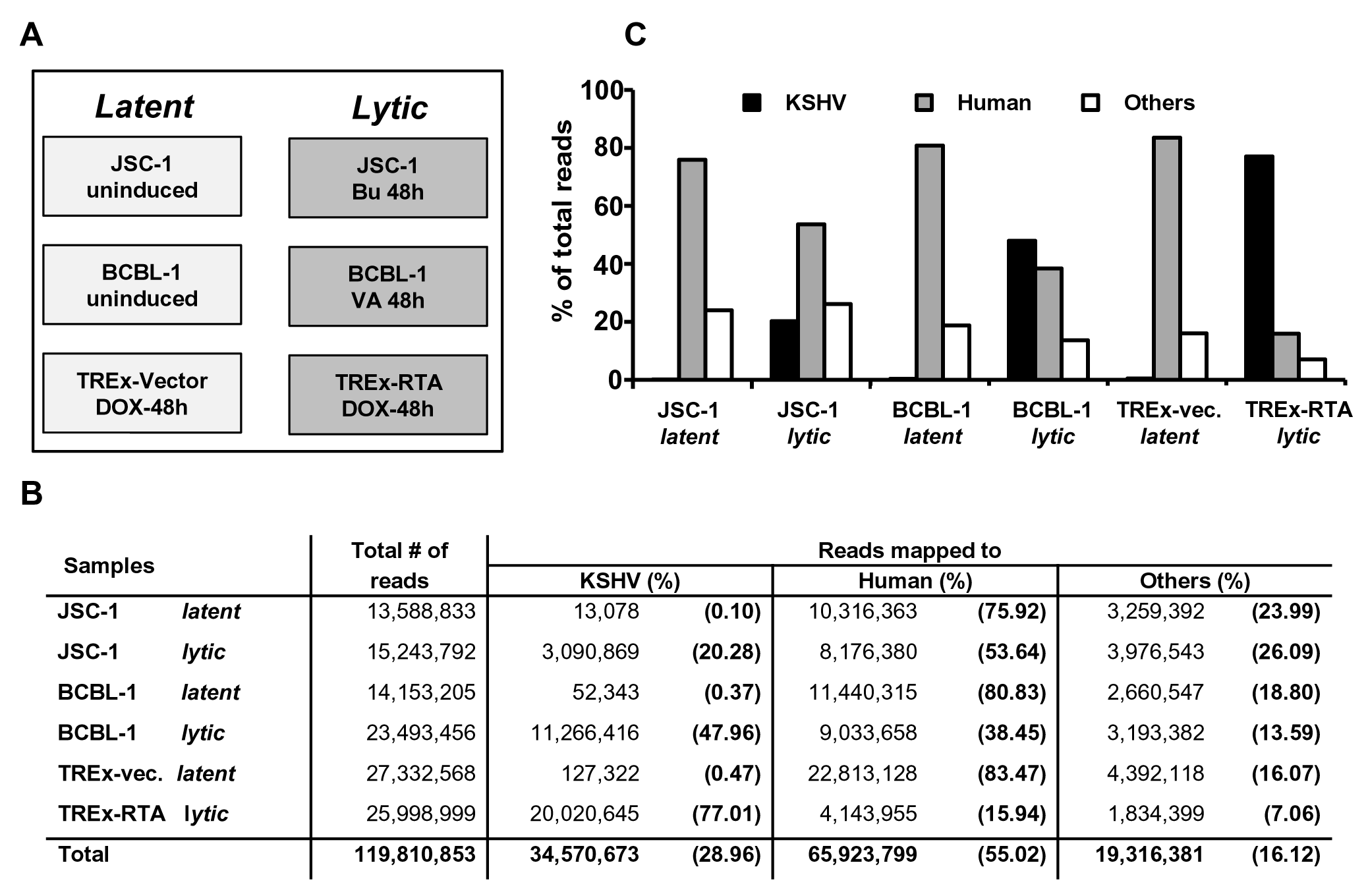

Supplement: Figure S1 — PA-seq analysis of KSHV transcripts. (A) Three KSHV-infected PEL (primary effusion lymphoma)-derived B-cell lines were used in PA-seq analysis during virus latent infection (left column) or lytic infection (right column). (B) Total numbers of sequence reads from each sample mapped to KSHV genome (Genbank acc no U75698.1) or human genome (UCSC version hg19). The other unassigned reads including those mapped to EBV genome (Genbank acc no V01555.2) (3233 reads or 0.02% in latent and 1998744 reads or 13.11% in lytic infection of JSC-1 cells) are shown as others. (C) A bar graph depicting % distribution of the sequence reads from each sample assigned to KSHV or human genome or others unassigned reads. (TIF) [file ppat.1003749.s001.tif]

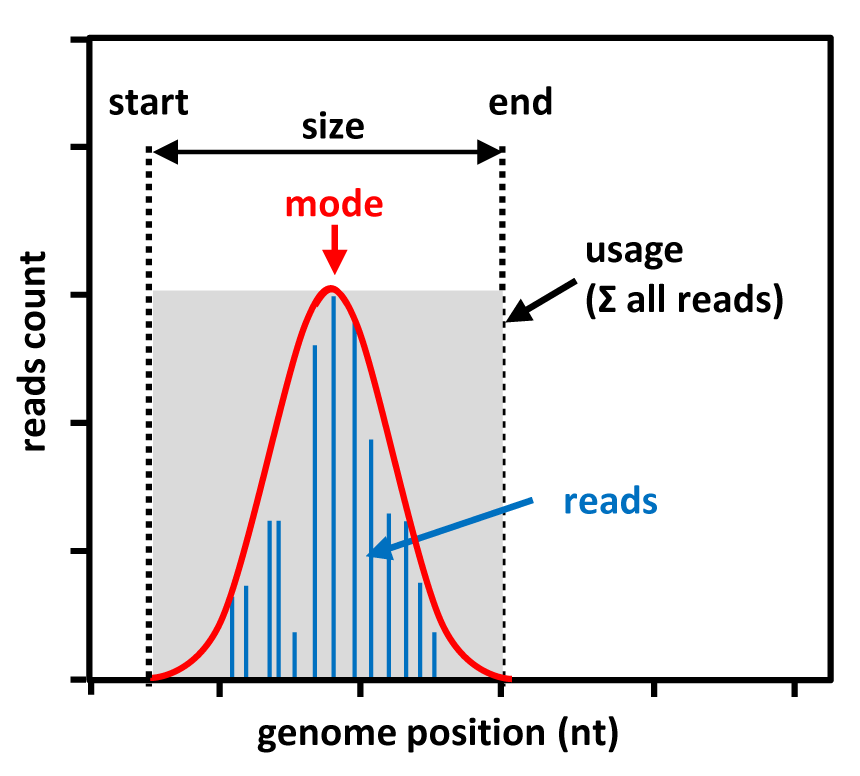

Supplement: Figure S2 — Determination of KSHV pA sites by F-seq analysis. Diagram shows PA peak (red line) identified by F-seq analysis of viral sequence reads (blue bars) aligned to the KSHV genome. The PA mode, a nucleotide position with the highest number of reads within the peak, was designated as a pA site. The peak size is a distance from nucleotide position of the beginning to the end of the peak within which a pA site is assigned. The total number of all reads within the peak represents usage of the pA site. (TIF) [file ppat.1003749.s002.tif]

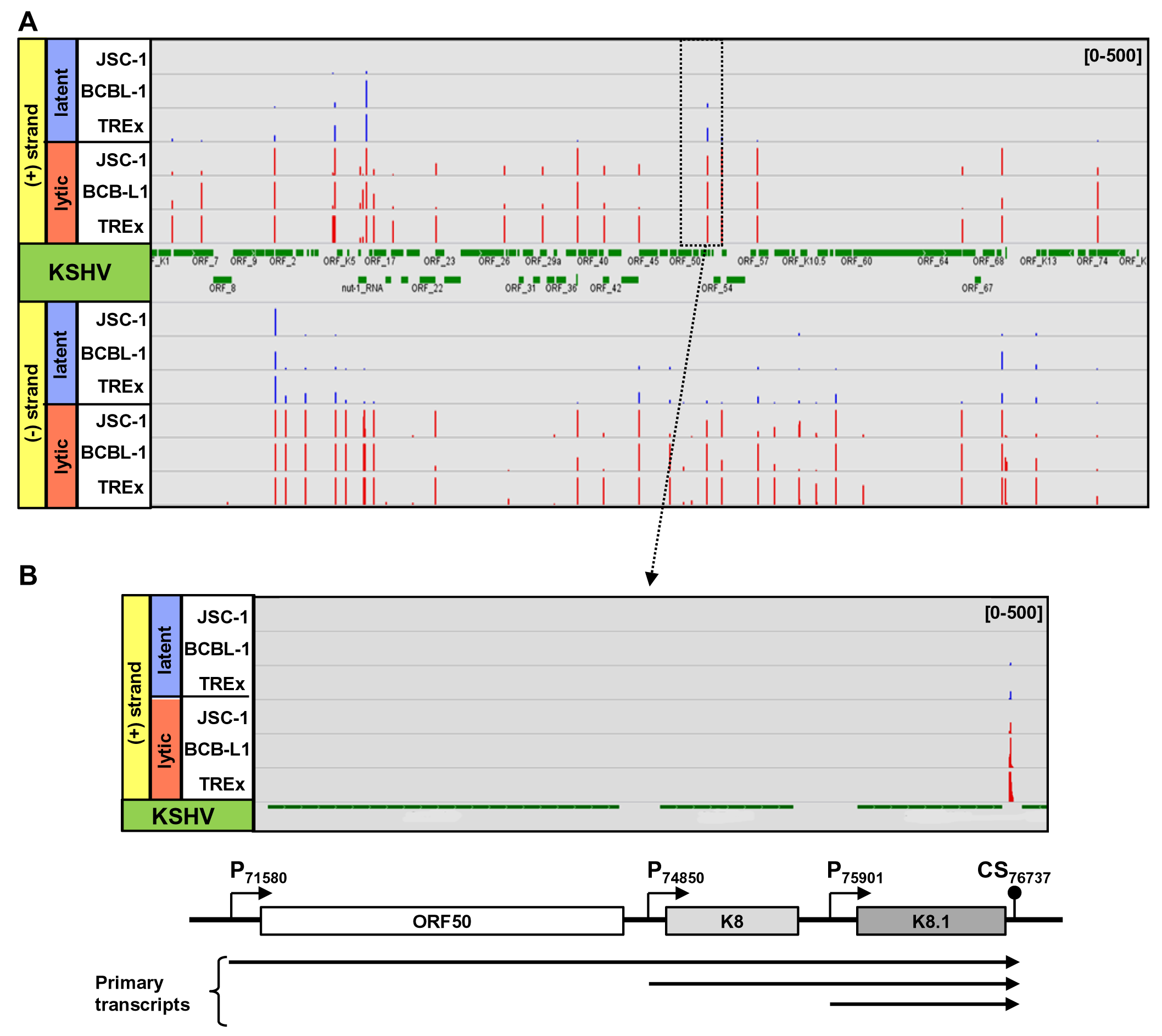

Supplement: Figure S3 — Visual distribution of KSHV-specific sequence reads obtained by PA-seq across viral genome. (A) Positions and frequency (scaled to maximal 500) of the sequence reads derived from B cells with latent (blue bars) or lytic (red bars) infection were visualized on KSHV genome by IGV software (http://www.broadinstitute.org/igv/). Green lines in the middle represent positions of reported KSHV genes. (B) A zoom-in to the locus containing ORF50 (RTA)-K8-K8.1 gene cluster where the sequence reads distribute in a plus (+) strand of the KSHV genome. Below is a diagram of previously reported gene structure and primary transcripts associated with this gene locus. Boxes represent an ORF with positions of mapped promoters (arrows) and a pA cleavage site (CS). The reads in latent infection represent spontaneous reactivation of this locus in a very small fraction of BCBL-1 cells and BCBL-1-derived TREx cells. (TIF) [file ppat.1003749.s003.tif]

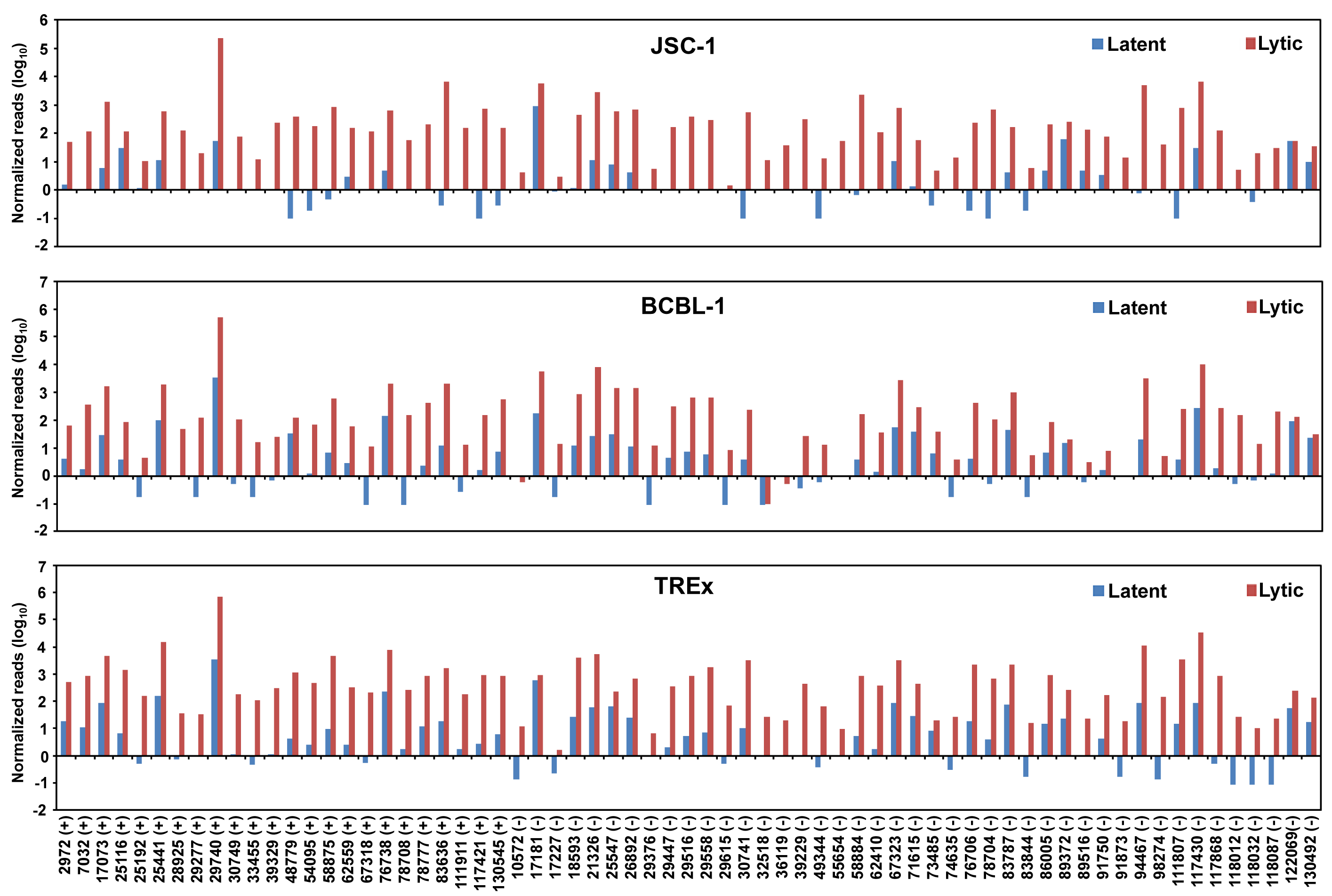

Supplement: Figure S4 — Illustration of pA site mapped to the KSHV genome in individual B cell lines with latent (blue) or lytic (red) KSHV infection. Scaled bars for each pA site represent normalized PA-seq reads per million. (TIF) [file ppat.1003749.s004.tif]

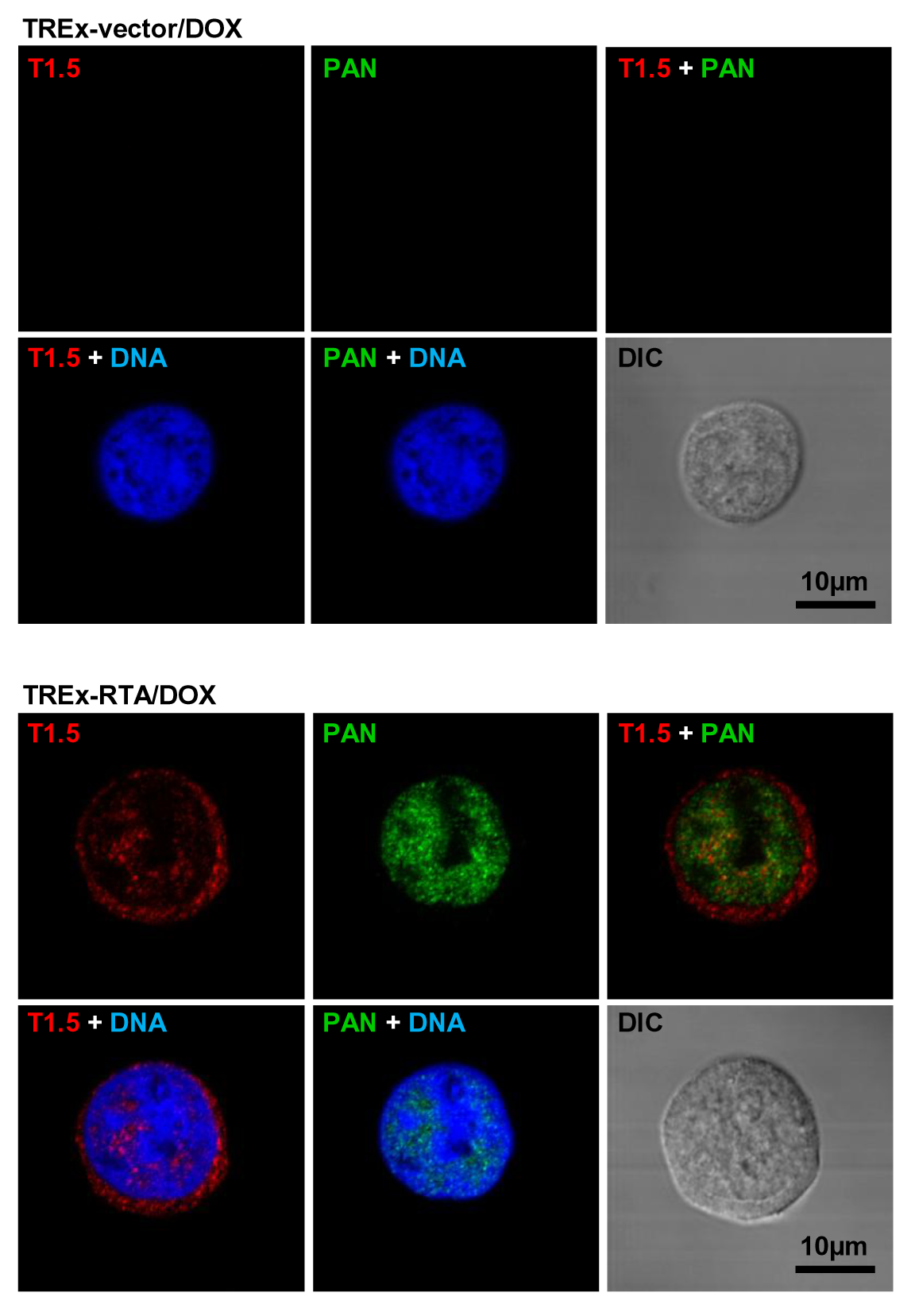

Supplement: Figure S5 — Localization of KSHV T1.5 and PAN lncRNAs in PEL cells. Specificity of each probe described in Figure 8 was tested in doxycycline-treated TREx cells by RNA FISH experiment as described in experimental procedures. The specific signal was observed only in TREx-RTA cells but not in TREx-vector cells. (TIF) [file ppat.1003749.s005.tif]
