# Supplementary material for: A Viral Genome Landscape of RNA Polyadenylation from KSHV Latent to Lytic Infection
Source: PLoS Pathog. 2013 Nov 14;9(11):e1003749. doi: 10.1371/journal.ppat.1003749 (PMC3828183; doi:10.1371/journal.ppat.1003749)
Supplement: Table S1 — Positions and strand specificity of all KSHV pA sites determined by F-seq analysis of combined six PA-seq libraries. (PDF) [file ppat.1003749.s006.pdf]

pA site (strand)	PA peak		
	Start	End	Usage
**2972 (+)**	2964	2982	14567
**7032 (+)**	7021	7045	29860
**17073 (+)**	17061	17099	163490
**25116 (+)**	25105	25139	38623
**25192 (+)**	25191	25198	4039
**25441 (+)**	25424	25472	425475
**28925 (+)**	28921	28929	3373
**29277 (+)**	29275	29282	3563
**29740 (+)**	29682	29780	29882191
**30749 (+)**	30743	30756	7324
**33455 (+)**	33452	33458	3028
**39329 (+)**	39324	39342	10781
**48779 (+)**	48761	48790	35939
**54095 (+)**	54088	54107	15154
**58875 (+)**	58862	58892	134808
**62559 (+)**	62553	62569	11015
**67318 (+)**	67313	67325	6793
**76738 (+)**	76723	76764	245562
**78708 (+)**	78702	78718	9997
**78777 (+)**	78764	78788	32236
**83636 (+)**	83619	83655	158045
**111911 (+)**	111904	111917	6560
**117421 (+)**	117411	117436	33623
**130545 (+)**	130534	130557	34135
**10572 (-)**	10568	10573	359
**17181 (-)**	17163	17200	229215
**17227 (-)**	17226	17229	387
**18593 (-)**	18576	18611	117252
**21326 (-)**	21307	21345	330954
**25547 (-)**	25529	25562	43067
**26892 (-)**	26876	26909	54254
**29376 (-)**	29367	29379	471
**29447 (-)**	29423	29461	17166
**29516 (-)**	29475	29531	39274
**29558 (-)**	29544	29585	61203
**29615 (-)**	29607	29625	1914
**30741 (-)**	30723	30761	87763
**32518 (-)**	32512	32523	768
**36119 (-)**	36114	36128	916
**39229 (-)**	39216	39243	14895
**49344 (-)**	49332	49352	2037
**55654 (-)**	55645	55658	858
**58884 (-)**	58863	58903	50571
**62410 (-)**	62399	62432	10982
**67323 (-)**	67306	67345	146698
**71615 (-)**	71598	71629	18081
**73485 (-)**	73478	73496	1577
**74635 (-)**	74628	74641	923
**76706 (-)**	76690	76725	64325
**78704 (-)**	78690	78723	27327
**83787 (-)**	83771	83808	78415
**83844 (-)**	83841	83848	571
**86005 (-)**	85990	86022	26076
**89372 (-)**	89358	89386	11103
**89516 (-)**	89506	89525	2254
**91750 (-)**	91739	91764	5230
**91873 (-)**	91868	91878	654
**94467 (-)**	94445	94487	401925
**98274 (-)**	98264	98286	3986
**111807 (-)**	111787	111824	98477
**117430 (-)**	117397	117458	1078460
**117868 (-)**	117818	117890	27010
**118012 (-)**	117997	118022	3893
**118032 (-)**	118026	118038	767
**118087 (-)**	118067	118102	4998
**122069(-)**	122055	122083	12021
**130492 (-)**	130470	130502	5036

Table S1
