# Supplementary material for: A Viral Genome Landscape of RNA Polyadenylation from KSHV Latent to Lytic Infection
Source: PLoS Pathog. 2013 Nov 14;9(11):e1003749. doi: 10.1371/journal.ppat.1003749 (PMC3828183; doi:10.1371/journal.ppat.1003749)
Supplement: Table S2 — The pA sites mapped by PA-seq in selected KSHV viral transcripts are comparable to the pA sites previously mapped by traditional methods. (PDF) [file ppat.1003749.s007.pdf]

Gene	Strand	pA site-position (nt)		Reference
		Reported	PA-seq	
**T1.5 RNA**	**+**	25440	25441	**[34)**
**PAN**	**+**	29741	29740	**[8]**
**ORF57**	**+**	83608	83636	**[39]**
**ORF45**	**-**	67325	67323	**[38]**
**ORF58**	**-**	94469/94477	94467	**[39]**
**K12**	**-**	117432	117430	**[40]**
**K13**	**-**	122060	122069	**[41]**
**ORF74**	**-**	130545	130545	**[42]**

Table S2
