# Supplementary material for: A Viral Genome Landscape of RNA Polyadenylation from KSHV Latent to Lytic Infection
Source: PLoS Pathog. 2013 Nov 14;9(11):e1003749. doi: 10.1371/journal.ppat.1003749 (PMC3828183; doi:10.1371/journal.ppat.1003749)
Supplement: Table S3 — Utilization of identified pA site with individual or cluster of KSHV genes. Adjacent 3′UTR length calculated as a distance between mapped KSHV sites to an immediately upstream KSHV ORF. N/A-not applicable. (PDF) [file ppat.1003749.s008.pdf]

pA site (strand)	Gene/gene cluster	# of genes/pA	Adjacent gene	3UTR
**2972 (+)**	K1, ORF4	2	ORF4	178
**7032 (+)**	ORF6	1	ORF6	421
**17073 (+)**	ORF7, 8, 9, 10, 11	5	ORF11	60
**25116 (+)**	T1.5	1	T1.5	N/A
**25192 (+)**	T1.5	1	T1.5	N/A
**25441 (+)**	T1.5	1	T1.5	N/A
**28925 (+)**	K7, PAN, T6.1	3	PAN	N/A
**29277 (+)**	K7, PAN, T6.1	3	K7	275
**29740 (+)**	K7, PAN, T6.1	3	K7	738
**30749 (+)**	ORF16	1	ORF16	77
**33455 (+)**	ORF18	1	ORF18	258
**39329 (+)**	ORF21, 22	2	ORF22	24
**48779 (+)**	ORF25, 26, 27	3	ORF27	34
**54095 (+)**	ORF28, 30, 31, 32, 33	5	ORF33	396
**58875 (+)**	ORF34, 35, 36, 37, 38	5	ORF38	2
**62559 (+)**	ORF40, 41	2	ORF41	115
**67318 (+)**	ORF44	1	ORF44	60
**76738 (+)**	ORF50, K8, K8.1	3	K8.1	43
**78708 (+)**	ORF54	1	ORF54	85
**78777 (+)**	ORF54	1	ORF54	154
**83636 (+)**	ORF56, 57	2	ORF57	92
**111911 (+)**	ORF63, 64	2	ORF64	4
**117421 (+)**	ORF68, 69	2	ORF69	75
**130545 (+)**	K14, ORF74, altRNA	3	ORF74	145
**10572 (-)**	N/A	0	N/A	N/A
**17181 (-)**	ORF2, K2	2	vIL6	80
**17227 (-)**	ORF2, K2	2	vIL6	34
**18593 (-)**	ORF70, K3	2	K3	15
**21326 (-)**	K4.2, K4.1, K4	3	K4	222
**25547 (-)**	K5	1	K5	166
**26892 (-)**	K6	1	K6	245
**29376 (-)**	N/A	0	vnct rep	N/A
**29447 (-)**	N/A	0	vntc rep	N/A
**29516 (-)**	N/A	0	vnct rep	N/A
**29558 (-)**	N/A	0	vntc rep	N/A
**29615 (-)**	N/A	0	vnct rep	N/A
**30741 (-)**	ORF17	1	ORF17	80
**32518 (-)**	ORF20, 19	2	ORF19	676
**36119 (-)**	N/A	0	N/A	N/A
**39229 (-)**	ORF24, 23	2	ORF23	73
**49344 (-)**	ORF29a, 29	2	ORF29	18
**55654 (-)**	N/A	0	N/A	N/A
**58884 (-)**	ORF39	1	ORF39	89
**62410 (-)**	ORF43, 42	2	ORF42	26
**67323 (-)**	ORF48, 47, 46, 45	4	ORF45	30
**71615 (-)**	asORF50S, ORF49	2	ORF49	15
**73485 (-)**	asORF50S	1	asORF50S	N/A
**74635 (-)**	N/A	0	N/A	N/A
**76706 (-)**	ORF53, 52	2	ORF52	96
**78704 (-)**	ORF55	1	ORF55	61
**83787 (-)**	K9	1	K9	73
**83844 (-)**	K9	1	K9 alt	16
**86005 (-)**	K10.1, K10	2	K10	69
**89372 (-)**	K10.6, K10.5	2	K10.5	228
**89516 (-)**	K10.6, K10.5	2	K10.5 alt	84
**91750 (-)**	K11.1, K11	2	K11	214
**91873 (-)**	K11.1, K11	2	K11 alt	91
**94467 (-)**	ORF62, 61, 60, 59, 58	5	ORF58	4
**98274 (-)**	ORF62	1	ORF62	1925
**111807 (-)**	ORF67, 66, 65	3	ORF65	124
**117430 (-)**	K12	1	K12	489
**117868 (-)**	K12	1	K12	51
**118012 (-)**	N/A	0	K12 internal	N/A
**118032 (-)**	N/A	0	K12 internal	N/A
**118087 (-)**	N/A	0	K12 internal	N/A
**122069(-)**	ORF73, 12, K13	3	K13	76
**130492 (-)**	K15, ORF75	2	ORF75	59
	**Median**	**2**	**Median**	**80**

Table S3
