# Supplementary material for: A Viral Genome Landscape of RNA Polyadenylation from KSHV Latent to Lytic Infection
Source: PLoS Pathog. 2013 Nov 14;9(11):e1003749. doi: 10.1371/journal.ppat.1003749 (PMC3828183; doi:10.1371/journal.ppat.1003749)
Supplement: Table S4 — KSHV genes contain alternative pA sites which can be used during virus infection. Individual pA site usage (%) was calculated from total number of sequence reads for all pA sites in a given gene transcript. (PDF) [file ppat.1003749.s009.pdf]

Gene (total reads)	pA sites	Reads count	Use (%)
**T1.5 (468137)**	25116 (+)	38623	**8.2**
	25192 (+)	4039	**0.9**
	25441 (+)	425475	**90.9**
**PAN (29889127)**	28925 (+)	3373	**<0.1**
	29277 (+)	3563	**<0.1**
	29740 (+)	29882191	**100.0**
**ORF54 (42233)**	78708 (+)	9997	**23.7**
	78777 (+)	32236	**76.3**
**vIL6 (229602)**	17181 (-)	229215	**99.8**
	17227 (-)	387	**0.2**
**vnct rep (120028)**	29376 (-)	471	**0.4**
	29447 (-)	17166	**14.3**
	29516 (-)	39274	**32.7**
	29558 (-)	61203	**51.0**
	29615 (-)	1914	**1.6**
**K9 (78986)**	83787 (-)	78415	**99.3**
	83844 (-)	571	**0.7**
**K10.5 (13357)**	89372 (-)	11103	**83.1**
	89516 (-)	2254	**16.9**
**K11 (5884)**	91750 (-)	5230	**88.9**
	91873 (-)	654	**11.1**
**K12 (1105470)**	117430 (-)	1078460	**97.6**
	117868 (-)	27010	**2.4**
**K12 internal (9656)**	118012 (-)	3893	**40.3**
	118032 (-)	767	**7.9**
	118087 (-)	4998	**51.8**

Table S4
