# Supplementary material for: A Viral Genome Landscape of RNA Polyadenylation from KSHV Latent to Lytic Infection
Source: PLoS Pathog. 2013 Nov 14;9(11):e1003749. doi: 10.1371/journal.ppat.1003749 (PMC3828183; doi:10.1371/journal.ppat.1003749)
Supplement: Table S5 — Normalized pA site reads mapped to the KSHV genome in individual B cell lines with latent or lytic KSHV infection. (PDF) [file ppat.1003749.s010.pdf]

pA site	Usage (normalized to million mapped reads)					
	JSC-1		BCBL-1		TREx BCBL-1	
	Latent	Lytic	Latent	Lytic	Latent	Lytic
**2972 (+)**	1.5	49.9	4.1	66.8	18.4	503.4
**7032 (+)**	0.0	114.1	1.7	369.0	11.4	860.8
**17073 (+)**	5.9	1305.2	29.3	1626.6	90.1	4688.7
**25116 (+)**	31.5	121.7	3.9	88.6	6.9	1445.3
**25192 (+)**	1.2	10.7	0.2	4.5	0.5	157.3
**25441 (+)**	11.5	618.8	98.8	1872.8	157.0	15544.6
**28925 (+)**	0.0	131.1	0.0	49.7	0.7	36.0
**29277 (+)**	0.0	19.7	0.2	125.2	0.0	33.0
**29740 (+)**	52.5	227575.2	3331.2	503546.4	3569.6	702486.3
**30749 (+)**	0.0	78.3	0.5	105.8	1.1	176.4
**33455 (+)**	0.0	12.0	0.2	16.3	0.5	105.5
**39329 (+)**	0.0	241.1	0.7	25.5	1.1	311.0
**48779 (+)**	0.1	397.3	34.0	126.8	4.3	1190.5
**54095 (+)**	0.2	184.3	1.2	68.0	2.6	481.0
**58875 (+)**	0.5	861.9	6.7	602.2	9.6	4658.5
**62559 (+)**	2.9	154.8	2.9	59.6	2.5	328.6
**67318 (+)**	0.0	118.5	0.1	11.3	0.6	215.8
**76738 (+)**	4.8	664.0	146.6	2026.0	225.2	7864.9
**78708 (+)**	0.0	59.2	0.1	150.9	1.8	257.6
**78777 (+)**	0.0	202.3	2.3	430.9	12.0	865.3
**83636 (+)**	0.3	6757.4	12.0	2023.6	18.9	1665.9
**111911 (+)**	0.0	155.4	0.3	13.1	1.8	186.1
**117421 (+)**	0.1	730.7	1.6	158.6	2.7	914.2
**130545 (+)**	0.3	152.5	7.7	561.4	6.4	860.1
**10572 (-)**	0.0	4.3	0.0	0.6	0.1	12.2
**17181 (-)**	914.3	5975.2	177.2	5590.4	607.2	951.6
**17227 (-)**	0.9	3.0	0.2	14.7	0.2	1.6
**18593 (-)**	1.2	444.8	12.2	841.2	26.7	3906.5
**21326 (-)**	11.8	2917.6	26.9	8330.1	61.9	5260.9
**25547 (-)**	8.0	587.9	31.6	1436.7	66.4	219.6
**26892 (-)**	4.3	685.2	11.1	1457.9	24.5	670.5
**29376 (-)**	0.0	5.5	0.1	12.0	0.0	6.8
**29447 (-)**	0.0	164.1	4.4	320.3	2.1	360.7
**29516 (-)**	0.0	401.6	7.5	656.6	5.5	877.6
**29558 (-)**	0.0	294.3	6.1	668.7	7.1	1824.1
**29615 (-)**	0.0	1.5	0.1	8.3	0.5	71.0
**30741 (-)**	0.1	556.4	3.7	231.9	10.3	3166.1
**32518 (-)**	0.0	11.5	0.1	0.1	0.0	26.3
**36119 (-)**	0.0	38.3	0.0	0.5	0.0	19.6
**39229 (-)**	0.0	313.6	0.3	28.0	1.0	445.6
**49344 (-)**	0.1	13.5	0.6	13.6	0.4	65.9
**55654 (-)**	0.0	53.3	0.0	1.0	0.0	9.8
**58884 (-)**	0.7	2352.9	3.9	160.1	5.4	853.8
**62410 (-)**	0.0	110.2	1.4	35.5	1.7	371.0
**67323 (-)**	10.6	827.8	54.9	2840.3	85.2	3187.2
**71615 (-)**	1.4	59.5	39.5	287.0	29.7	431.8
**73485 (-)**	0.3	5.0	6.6	38.2	8.5	19.4
**74635 (-)**	0.0	14.5	0.2	3.8	0.3	27.9
**76706 (-)**	0.2	233.0	4.3	433.5	19.2	2168.8
**78704 (-)**	0.1	702.7	0.5	107.7	3.9	708.7
**83787 (-)**	4.2	165.2	44.6	1031.4	77.7	2204.9
**83844 (-)**	0.2	6.1	0.2	5.5	0.2	15.8
**86005 (-)**	4.7	208.7	7.0	87.3	15.1	888.7
**89372 (-)**	62.7	264.7	15.3	20.9	23.9	261.7
**89516 (-)**	4.9	138.0	0.6	3.1	1.0	23.0
**91750 (-)**	3.4	77.7	1.6	8.2	4.4	167.0
**91873 (-)**	0.0	14.4	0.0	1.1	0.2	19.3
**94467 (-)**	0.8	5101.4	19.9	3161.5	88.8	11504.2
**98274 (-)**	0.0	40.6	0.0	5.0	0.1	141.7
**111807 (-)**	0.1	781.9	3.9	256.0	14.5	3479.9
**117430 (-)**	31.3	6753.4	268.6	9848.0	86.7	32984.4
**117868 (-)**	0.0	129.0	1.9	268.0	0.5	831.0
**118012 (-)**	0.0	5.1	0.5	155.9	0.1	27.4
**118032 (-)**	0.4	20.2	0.7	13.9	0.1	10.1
**118087 (-)**	0.0	30.7	1.2	200.1	0.1	23.8
**122069(-)**	53.4	56.0	90.9	136.0	56.1	237.7
**130492 (-)**	9.6	35.9	22.9	32.2	17.6	133.0

Table S5
