# Supplementary material for: A Viral Genome Landscape of RNA Polyadenylation from KSHV Latent to Lytic Infection
Source: PLoS Pathog. 2013 Nov 14;9(11):e1003749. doi: 10.1371/journal.ppat.1003749 (PMC3828183; doi:10.1371/journal.ppat.1003749)
Supplement: Table S6 — The usage of individual KSHV pA sites during latent and lytic infection from combined datasets of three PEL cell lines (Table S5). The fold increase for each pA site was calculated by dividing the sequence reads from lytic samples by number of the reads from latency. N/A-not applicable. (PDF) [file ppat.1003749.s011.pdf]

pA site (strand)	Usage (normalized to million mapped reads)				Fold (lytic /latent)
	Latent	Lytic	Latent (log10)	Lytic (log10)	
**2972 (+)**	24.0	620.1	1.4	2.8	25.8
**7032 (+)**	13.2	1343.9	1.1	3.1	102.1
**17073 (+)**	125.3	7620.5	2.1	3.9	60.8
**25116 (+)**	42.3	1655.6	1.6	3.2	39.2
**25192 (+)**	1.9	172.5	0.3	2.2	92.8
**25441 (+)**	267.4	18036.1	2.4	4.3	67.5
**28925 (+)**	0.7	216.8	-0.1	2.3	292.6
**29277 (+)**	0.2	177.9	-0.8	2.3	1022.3
**29740 (+)**	6953.2	1433608.0	3.8	6.2	206.2
**30749 (+)**	1.7	360.5	0.2	2.6	217.7
**33455 (+)**	0.7	133.8	-0.2	2.1	204.7
**39329 (+)**	1.8	577.5	0.3	2.8	315.7
**48779 (+)**	38.4	1714.7	1.6	3.2	44.6
**54095 (+)**	4.0	733.2	0.6	2.9	184.1
**58875 (+)**	16.8	6122.5	1.2	3.8	364.1
**62559 (+)**	8.3	543.0	0.9	2.7	65.4
**67318 (+)**	0.7	345.6	-0.2	2.5	528.7
**76738 (+)**	376.7	10554.8	2.6	4.0	28.0
**78708 (+)**	1.9	467.7	0.3	2.7	249.5
**78777 (+)**	14.2	1498.4	1.2	3.2	105.2
**83636 (+)**	31.2	10446.8	1.5	4.0	335.1
**111911 (+)**	2.1	354.6	0.3	2.5	169.5
**117421 (+)**	4.3	1803.5	0.6	3.3	417.3
**130545 (+)**	14.4	1573.9	1.2	3.2	109.6
**10572 (-)**	0.1	17.1	-0.9	1.2	130.8
**17181 (-)**	1698.7	12517.2	3.2	4.1	7.4
**17227 (-)**	1.3	19.3	0.1	1.3	15.3
**18593 (-)**	40.0	5192.5	1.6	3.7	129.7
**21326 (-)**	100.6	16508.6	2.0	4.2	164.0
**25547 (-)**	106.1	2244.3	2.0	3.4	21.2
**26892 (-)**	39.9	2813.6	1.6	3.4	70.4
**29376 (-)**	0.1	24.3	-1.1	1.4	279.4
**29447 (-)**	6.5	845.1	0.8	2.9	129.4
**29516 (-)**	13.0	1935.8	1.1	3.3	148.7
**29558 (-)**	13.2	2787.1	1.1	3.4	210.5
**29615 (-)**	0.6	80.8	-0.2	1.9	132.4
**30741 (-)**	14.1	3954.3	1.2	3.6	279.9
**32518 (-)**	0.1	37.9	-1.1	1.6	435.7
**36119 (-)**	0.0	58.4	N/A	1.8	N/A
**39229 (-)**	1.4	787.1	0.1	2.9	582.8
**49344 (-)**	1.1	93.0	0.0	2.0	84.7
**55654 (-)**	0.0	64.1	N/A	1.8	N/A
**58884 (-)**	10.0	3366.9	1.0	3.5	335.3
**62410 (-)**	3.1	516.7	0.5	2.7	164.8
**67323 (-)**	150.7	6855.3	2.2	3.8	45.5
**71615 (-)**	70.6	778.3	1.8	2.9	11.0
**73485 (-)**	15.4	62.6	1.2	1.8	4.1
**74635 (-)**	0.5	46.2	-0.3	1.7	96.3
**76706 (-)**	23.6	2835.3	1.4	3.5	120.0
**78704 (-)**	4.5	1519.2	0.7	3.2	334.5
**83787 (-)**	126.4	3401.4	2.1	3.5	26.9
**83844 (-)**	0.5	27.4	-0.3	1.4	50.6
**86005 (-)**	26.9	1184.8	1.4	3.1	44.1
**89372 (-)**	101.9	547.3	2.0	2.7	5.4
**89516 (-)**	6.5	164.1	0.8	2.2	25.1
**91750 (-)**	9.4	252.8	1.0	2.4	27.0
**91873 (-)**	0.2	34.7	-0.8	1.5	199.3
**94467 (-)**	109.5	19767.1	2.0	4.3	180.6
**98274 (-)**	0.1	187.3	-0.9	2.3	1432.4
**111807 (-)**	18.5	4517.8	1.3	3.7	243.8
**117430 (-)**	386.5	49585.8	2.6	4.7	128.3
**117868 (-)**	2.4	1228.1	0.4	3.1	503.9
**118012 (-)**	0.6	188.5	-0.2	2.3	309.3
**118032 (-)**	1.2	44.2	0.1	1.6	37.7
**118087 (-)**	1.3	254.6	0.1	2.4	195.0
**122069(-)**	200.5	429.7	2.3	2.6	2.1
**130492 (-)**	50.1	201.0	1.7	2.3	4.0
**Total**	**11303.20**	**1648659.57**			**145.86**

Table S6
