# Supplementary material for: A Viral Genome Landscape of RNA Polyadenylation from KSHV Latent to Lytic Infection
Source: PLoS Pathog. 2013 Nov 14;9(11):e1003749. doi: 10.1371/journal.ppat.1003749 (PMC3828183; doi:10.1371/journal.ppat.1003749)
Supplement: Table S7 — Classification of KSHV pA sites based on the PA peak size. The size of each peak was calculated as a distance between start and end of the peak and it was correlated with pA site usage (Table S1). Based on their peak size, all pA sites were divided into three categories: narrow (NP, ≤30 nts), broad (BP, >30, ≤45 nts) or wide (WP, >45 nts) peaks. (PDF) [file ppat.1003749.s012.pdf]

pA site (strand)	PA peak size/usage				Category
	size (nt)	size (log_2)	usage	usage (log_2)	
2972 (+)	18	4.2	14567	13.8	NP
7032 (+)	24	4.6	29860	14.9	NP
17073 (+)	38	5.2	163490	17.3	BP
25116 (+)	34	5.1	38623	15.2	BP
25192 (+)	7	2.8	4039	12.0	NP
25441 (+)	48	5.6	425475	18.7	WP
28925 (+)	8	3.0	3373	11.7	NP
29277 (+)	7	2.8	3563	11.8	NP
29740 (+)	98	6.6	29882191	24.8	WP
30749 (+)	13	3.7	7324	12.8	NP
33455 (+)	6	2.6	3028	11.6	NP
39329 (+)	18	4.2	10781	13.4	NP
48779 (+)	29	4.9	35939	15.1	NP
54095 (+)	19	4.2	15154	13.9	NP
58875 (+)	30	4.9	134808	17.0	NP
62559 (+)	16	4.0	11015	13.4	NP
67318 (+)	12	3.6	6793	12.7	NP
76738 (+)	41	5.4	245562	17.9	BP
78708 (+)	16	4.0	9997	13.3	NP
78777 (+)	24	4.6	32236	15.0	NP
83636 (+)	36	5.2	158045	17.3	BP
111911 (+)	13	3.7	6560	12.7	NP
117421 (+)	25	4.6	33623	15.0	NP
130545 (+)	23	4.5	34135	15.1	NP
10572 (-)	5	2.3	359	8.5	NP
17181 (-)	37	5.2	229215	17.8	BP
17227 (-)	3	1.6	387	8.6	NP
18593 (-)	35	5.1	117252	16.8	BP
21326 (-)	38	5.2	330954	18.3	BP
25547 (-)	33	5.0	43067	15.4	BP
26892 (-)	33	5.0	54254	15.7	BP
29376 (-)	12	3.6	471	8.9	NP
29447 (-)	38	5.2	17166	14.1	BP
29516 (-)	56	5.8	39274	15.3	WP
29558 (-)	41	5.4	61203	15.9	BP
29615 (-)	18	4.2	1914	10.9	NP
30741 (-)	38	5.2	87763	16.4	BP
32518 (-)	11	3.5	768	9.6	NP
36119 (-)	14	3.8	916	9.8	NP
39229 (-)	27	4.8	14895	13.9	NP
49344 (-)	20	4.3	2037	11.0	NP
55654 (-)	13	3.7	858	9.7	NP
58884 (-)	40	5.3	50571	15.6	BP
62410 (-)	33	5.0	10982	13.4	BP
67323 (-)	39	5.3	146698	17.2	BP
71615 (-)	31	5.0	18081	14.1	BP
73485 (-)	18	4.2	1577	10.6	NP
74635 (-)	13	3.7	923	9.9	NP
76706 (-)	35	5.1	64325	16.0	BP
78704 (-)	33	5.0	27327	14.7	BP
83787 (-)	37	5.2	78415	16.3	BP
83844 (-)	7	2.8	571	9.2	NP
86005 (-)	32	5.0	26076	14.7	BP
89372 (-)	28	4.8	11103	13.4	NP
89516 (-)	19	4.2	2254	11.1	NP
91750 (-)	25	4.6	5230	12.4	NP
91873 (-)	10	3.3	654	9.4	NP
94467 (-)	42	5.4	401925	18.6	BP
98274 (-)	22	4.5	3986	12.0	NP
111807 (-)	37	5.2	98477	16.6	BP
117430 (-)	61	5.9	1078460	20.0	WP
117868 (-)	72	6.2	27010	14.7	WP
118012 (-)	25	4.6	3893	11.9	NP
118032 (-)	12	3.6	767	9.6	NP
118087 (-)	35	5.1	4998	12.3	BP
122069(-)	28	4.8	12021	13.6	NP
130492 (-)	32	5.0	5036	12.3	BP

Table S7
