# Supplementary material for: A Viral Genome Landscape of RNA Polyadenylation from KSHV Latent to Lytic Infection
Source: PLoS Pathog. 2013 Nov 14;9(11):e1003749. doi: 10.1371/journal.ppat.1003749 (PMC3828183; doi:10.1371/journal.ppat.1003749)
Supplement: Table S8 — Frequency of pA site usage in correlation to PA peak size. The Pearson (r) and Spearman (rs) correlation coefficients expressing correlation between peak size and number of reads were calculated for each group of pA site. (PDF) [file ppat.1003749.s013.pdf]

PA peaks	All (n=67)	Narrow (n=38)	Broad (n=24)	Wide (n=5)
**Peak size (median, nt)**	27	17	36.5	61
**Reads count (median)**	14,895	4,013	62,764	425,475
**Pearson coefficient (r)**	0.55	0.53	0.63	0.89
**Spearman coefficient (r_s)**	0.86	0.73	0.65	0.3

Table S8
