# Supplementary material for: A Viral Genome Landscape of RNA Polyadenylation from KSHV Latent to Lytic Infection
Source: PLoS Pathog. 2013 Nov 14;9(11):e1003749. doi: 10.1371/journal.ppat.1003749 (PMC3828183; doi:10.1371/journal.ppat.1003749)
Supplement: Table S9 — Canonical and non-canonical PAS (red) detected in a region covering 50 nts immediately upstream of the mapped pA site. N/D-not detectable. (PDF) [file ppat.1003749.s014.pdf]

pA site (strand)	Sequence 50nt upstream of mapped pA site, PAS	PAS
2972 (+)	UGUGGUAUGUAUUGUACUAAAGAUACCGACCAAUACAAGACAACUAAUAU	AAUACA
7032 (+)	UGGGCCUUGGAGUUAAAGCUGUCACUAAUAAACGACGUUGAAAUUUUCUU	AAUAAA
17073 (+)	UCCCACCCACACAUUUGUCUUUAUUGCUUUCAAAUAAAACGGUGUUCUGU	AAUAAA
25116 (+)	GUCAAAAAACUUUAAAAACAUCGUUAUCCAAUAUCAUUAAAAACCACACC	AUUAAA
25192 (+)	GUGUUAGUGUCACCCACUGUACACAAGGCGUGUCGUAUAUGUAGUAUAGG	N/D
25441 (+)	CGGCUAUUCUGUCCCAGCAUAGGCUCUUGAAAUAAACAAUGUUUACCGAG	AAUAAA
28925 (+)	CGCGGUCUGGAGCUCUCCAUAAGCCCGCAGAACAAAAGCUGCGAUUUGCC	N/D
29277 (+)	GUGUAUCUUAUUGGUGCGUUGUGAAGCAUUUUAAAAUGCGUUUUAGAUUG	N/D
29740 (+)	UACCAUAACGUAAUGCAACUUACAACAUAAAUAAAGGUCAAUGUUUAAUC	AAUAAA
30749 (+)	UAGAGGGUUGCAUGUUAUACAAACAACAUAAACAUUAAAUGAACAUUGUU	CAUAAA
33455 (+)	UGCUGGUUCAGGAUUUCGAAUAUCUCAUUAAACCUACUGCCUGUCAGAUU	AUUAAA
39329 (+)	UACAGACUGUUUUCCAUCCUUUAUUAGACGGUCAAUAAAGCGUAGAUUUU	AAUAAA
48779 (+)	UUCUAAAUUUUAAAUAAAGGUGUGUCACUGGUUACACCACGAUUAAAAAC	AAUAAA
54095 (+)	GUCUGUAAAACACUGUUUGGAGUUUCAAAUAAACCGAAGUACUGCUUAAA	AAUAAA
58875 (+)	AUAUCCCGCAUUAAAAAAGAAGAAAAAAAAUAAAGAAGCAAUUUAUUAAG	AAUAAA
62559 (+)	UUCUCCAGGUGCUUGGUAAAGAUGGUAUCACAAUAAAAAAUGUUUACUGG	AAUAAA
67318 (+)	CUCUCUUAGGACACUGAUGUGUUUGGGAAUAAAGCAUGAGACUUGACACC	AAUAAA
76738 (+)	UAGUGUAACACAAAACCAUAAAAGUAAAUAAACGUGUUUAUUGUUCACAU	AAUAAA
78708 (+)	AUUCGAUCCCGCAACCCACAGCAUCCCCCAAAUAAAAAAACGAGUGUACA	AAUAAA
78777 (+)	UGUUCGAUUCAUUACUGGUACCAGAGAAUAAAGCCAACCUAUGUCGAACC	AAUAAA
83636 (+)	GUAACUCACGUAGCCUUUCUCUAAUAAACAAGCUACCUGCAAACUAUACA	AAUAAA
111911 (+)	CGUCCCUGGCGGAGACGGUUGACAGAAUAAAGAAGUGGUACUUGUGACUC	AAUAAA
117421 (+)	GCUCCUAUUGCAACUGACCAUGUUCAGGUGGUAAUAAAGUCAUUAAACGA	AAUAAA
130545 (+)	CUAUGUAUUUGUUUUAUUAUGCGAUUAAAUGAGGGGUCUGAUCCCAAAAG	AUUAAA
10572 (-)	GCUCGAUGGCCUUGAAGUCUAUGUUUUGAAUAAAGGAUAGAUUCAGGGCG	AAUAAA
17181 (-)	CACUAAUAUACUUUGCGUGUCUAGACAAUAAAGAUUAACUGUCUACAGUC	AAUAAA
17227 (-)	ACGUCCACGAUAAGUAAAGAGCGACGUGACAUAAAAUUUCUUAUAACACU	CAUAAA
18593 (-)	CGUUCGGCUGUUUCGUCUGCCCUUAUGUUUCAUUAAAAGUAUAUAAGAGC	AUUAAA
21326 (-)	ACUCUACUGCACAAAAUUGUCUUGUAAAAAUAAAUGUGAAUGUCAGACAC	AAUAAA
25547 (-)	GGCCUGUGCCCAAAUCUGUCCUCAUUAAAAACAAGUAGCUGUUGACUUUC	AUUAAA
26892 (-)	GGGAUCCACUCAGGUGUUUUCCAAUAAAGCUGUUGUGGUUUUUCUCUCAU	AAUAAA
29376 (-)	GCUCCCAGCUGCCGCACACCACUUUAGUCCAAUGUUCUUACACGACUUUG	N/D
29447 (-)	AGCAAAACAUCAAAUAUUAGCCUGGAACUUCCAACACAACAUACACAUUA	N/D
29516 (-)	CCCAGCCAAAACACCGUUAUCAUUGUUACACAACGCUUUCACCUACAAGA	N/D
29558 (-)	GCCGUCUGGUCACUGGAGGUGUCCGGUGCGAACAAGGAAAAACCCAGCCA	N/D
29615 (-)	CGUUAAAUUGUCAAAAGUAUAACAUGUUUUUCCAAUAUACACUGGGAUAA	AAUAUA
30741 (-)	CUACCCUACUCCCCUGUUUGAAAAAAAAUAAACAUACGUUUUGAACAAUG	AAUAAA
32518 (-)	UGUACAUCCUGCAGAGAACCAAAUGAAUAAAACGCAGCUCUUGGGCGUGG	AAUAAA
36119 (-)	CCAUUACCCCCUCUAAGUAAAGCAAAUAAACAUUCCUGUAGUCCACGGUC	AAUAAA
39229 (-)	AAAGAAAGUAGAUAACCCCCGAGAACCCAAUAAAAGAGAGAAUUAGAAAC	AAUAAA
49344 (-)	AGACACAUGUACAAGCCCAUAUCCCCACAAUAAAAGCACAGCGCUCAAUU	AAUAAA
55654 (-)	CCUCCUGUUGAUGUUGGCCUCCAGAGCCGACUUAAUAAACUCUCUUUUAG	AAUAAA
58884 (-)	AUCCCCCGUUAUUUUUCACCCACCACGGAAUAAAAUACGUACAGAAAACC	AAUAAA
62410 (-)	CAUUGUUUCCCUUUUUUCAAAAUAAAGAUAAAAGCCUGGUCCAGGCCUCU	AAUAAA
67323 (-)	AACUGGCCGUGGCUGGACUGAUAAAUAAAAGAAUGGUGUCAAUACAGACC	AAUAAA
71615 (-)	CACCAUUGUAAACACGCAUUGUUCAGUAUACAAUAAAAAGGUCGAUCUUU	AAUAAA
73485 (-)	CCUGCCACGUACUAAUAAACGCCGGGUCUGUACCCGAUGGGUUAAAGGGG	AAUAAA
74635 (-)	GUUAAAAAUAAACUGCCUGGGAAGUUAACGCAGGCACAGACGCCCUUGCG	AAUAAA
76706 (-)	AAGAGUACCACUCUUUAUCAUGUGAACAAUAAACACGUUUAUUUACUUUU	AAUAAA
78704 (-)	UUCUCUGGUACCAGUAAUGAAUCGAACAAUAAUAAAAACAUUGGCUGUGU	AAUAAA
83787 (-)	UAUUGUUAUGGGAGGGAGUAUAUCAUAUCAAUAAAGAGACCAAAACAUUG	AAUAAA
83844 (-)	UAAUGCCGAAGCCGUUAUGGGAUGCCAUGCAAUAAAUAAUACAAAAUGCC	AAUAAA
86005 (-)	AAACCGGAUGUAAUCUUGUAGUCCAAAUAAACCAGACACGACAUUUCGCC	AAUAAA
89372 (-)	ACCUGUGAUUAUGUUAACCCGGAAUCGAUAAAUAAAAUAGAAAGCAUUUG	AAUAAA
89516 (-)	GCUGUGGUUAAGUGAAAGUGUGAGGAUAAAAUAAACACUACAUUUCACCC	AAUAAA
91750 (-)	CACUGAGUGGGACAUCAUUAAAGGUUAGCGCCACCGUGUGGCUGCAAAAU	AUUAAA
91873 (-)	CCUUUGUCUUACCGCCCUCCGUUUUGCACUAUAAAUAUCAUUGCCGUUAG	UAUAAA
94467 (-)	UAUGUAUCUCAUGUCUCCUGUCGGUAAUAAAUAAAGUUGUUGGCUAAUUG	AAUAAA
98274 (-)	UUGCUAAUAUAUCUUCCAAGGUCACCAAUAAAGAAGAGGUGUUGAGGCCC	AAUAAA
111807 (-)	AUAGGGCGGCCACUUUUUGUUUGAUGCGUCUAAUAAAACUAAUCAGUGUU	AAUAAA
117430 (-)	GUACAUUGCAGCGUGUGCCACAUGUUCAAAAACAAUAAACAGAUUAAAAG	AAUAAA
117868 (-)	AGCUAGCGUGCCCUCCCAAAGAGUGUCAGUAAAAUAAAAUACAAAAACAC	AAUAAA
118012 (-)	CCGGAUGUGUUACUAAAUGGGUGGCGCUGGAGGCUUGGGGCGAUACCACC	ACUAAA
118032 (-)	UGUUUGUGGCAGUUCAUGUCCCGGAUGUGUUACUAAAUGGGUGGCGCUGG	ACUAAA
118087 (-)	UGGUGCCCUCCUCCCUCCUCACUCCAAUCCCAAUGCAUGGAUAGAGGCUU	N/D
122069(-)	UCCGUAGAAUGUAUGUAUCUGAUUUAAUAAACACUAACAAGUUUUGUAAG	AAUAAA
130492 (-)	UUUGGGAUCAGACCCCUCAUUUAAUCGCAUAAUAAAACAAAUACAUAGUC	AAUAAA

Table S9
