# Supplementary material for: A Viral Genome Landscape of RNA Polyadenylation from KSHV Latent to Lytic Infection
Source: PLoS Pathog. 2013 Nov 14;9(11):e1003749. doi: 10.1371/journal.ppat.1003749 (PMC3828183; doi:10.1371/journal.ppat.1003749)
Supplement: Table S10 — Prevalence of canonical and non-canonical PAS 50 nts upstream of the mapped pA sites in all, narrow, broad, wide, top 10, and bottom 10 pA sites. (PDF) [file ppat.1003749.s015.pdf]

	PA peaks					
**PAS**	**All**	**Narrow**	**Broad**	**Wide**	**Top10**	**Bottom 10**
**AAUAAA**	46	24	18	4	10	6
**AUUAAA**	6	3	3	0	0	0
**ACUAAA**	2	2	0	0	0	1
**CAUAAA**	2	2	0	0	0	1
**AAUACA**	1	1	0	0	0	0
**AAUAUA**	1	1	0	0	0	0
**UAUAAA**	1	1	0	0	0	1
**N/D**	8	4	3	1	0	1
**Total**	**67**	**38**	**24**	**5**	**10**	**10**

Table S10
