# Supplementary material for: A Viral Genome Landscape of RNA Polyadenylation from KSHV Latent to Lytic Infection
Source: PLoS Pathog. 2013 Nov 14;9(11):e1003749. doi: 10.1371/journal.ppat.1003749 (PMC3828183; doi:10.1371/journal.ppat.1003749)
Supplement: Table S11 — Primers used in 3′RACE analyses. (PDF) [file ppat.1003749.s016.pdf]

Gene	pA site	Name	Position (nt)	Sequence	Product size (bp)*
**ORF27**	48779 (+)	oVM232	48454-48478	5’ -TCAGACCGCATCCCGTAACCACACT-3’	326
**ORF62**	98274 (-)	oVM236	98989-98665	5’ -TGTTCAGGGGTTGGCGGATGTGTTT-3’	416
**T1.5**	25116 (+)<br>25192 (+)<br>25441 (+)	oVM209	24907-24928	5’ -AACCTGTTGCCATGTATGGCGA-3’	210<br>286<br>535
**ORF54**	78708 (+)<br>78777 (+)	oVM233	78392-78416	5’ -CCAGATTGTGTTTGTGGAACGCAGA-3’	317<br>387
**K2**	17181 (+)<br>17227 (+)	oVM207	17693-17671	5’-GGGTATTCTAGAGCCCGCTGCTA-3’	513<br>467
**K11**	91750 (-)<br>91873 (-)	oVM235	92105-92081	5’ -CGTGGAGCGACATAATCGAGAACCT-3’	356<br>233
**K12**	117430 (-)<br>117868 (-)	oVM237	118128-118104	5’-CCTCCCTCCTCACTCCAATCCCAAT-3’	699<br>260
**vnct rep**	29376 (-)<br>29447 (-)<br>29516 (-)<br>29558 (-)<br>29615 (-)	oVM234	29770-29746	5’ -ACGCAAGTCAAGACACAAGTCAGGA-3’	395<br>324<br>255<br>213<br>156
**as-vIL6**	18200 (+)	oJGK22	17486-17504	5’-CTTCGTCAGAAGCTCCATG-3’	715
**as-ORF21**	36119 (-)	oVM247	36608-36584	5'-CTGGAGAGGGTGAGAATGGCGACCA-3	490
		oVM248**	36415-36391	5'-GCTGCATCATTCGCAGGATAGCGGT-3'	297
**as-ORF34**	55654 (-)	oVM250	56133-56111	5'-TGCCATGTGGACGCCCAGGGTCT-3'	480
		oVM251**	55937-55915	5'-GCGTCGACCAGGGCGTCAATGTC-3'	284
**as-K8**	74635 (-)	oVM253	75094-75070	5'-CCACTTTGGGAAGGCGCTGTAAGGT-3'	460
		oVM254**	74953-74929	5'-GGTTGCCCGTTGAGGCTTAGATCTT-3'	319

*** Actual 3’RACE products contain additional adaptor sequence (77 nts for primary and 55 nts for nested 3’RACE)**
**** Primers used in nested 3’RACE**

Table S11
